# Discovery of ancestral L-ornithine and L-lysine decarboxylases reveals parallel, pseudoconvergent evolution of polyamine biosynthesis

**DOI:** 10.1016/j.jbc.2021.101219

**Published:** 2021-09-21

**Authors:** Bin Li, Jue Liang, Colin C. Hanfrey, Margaret A. Phillips, Anthony J. Michael

**Affiliations:** 1 Department of Biochemistry, UT Southwestern Medical Center, Dallas, Texas, USA; 2 Institute of Food Research, Norwich, United Kingdom

**Keywords:** polyamine, ornithine, arginine, lysine, decarboxylase, putrescine, agmatine, cadaverine, AAT-fold, aspartate aminotransferase-fold, ADC, L-arginine decarboxylase, AIH, agmatine iminohydrolase, AR-fold, alanine racemase-fold, LDC, L-lysine decarboxylase, ODC, L-ornithine decarboxylase, O/LDC, bifunctional lysine/ornithine decarboxylase, NCPAH, N-carbamoylputrescine amidohydrolase, REC, receiver domain of bacterial response regulator

## Abstract

Polyamines are fundamental molecules of life, and their deep evolutionary history is reflected in extensive biosynthetic diversification. The polyamines putrescine, agmatine, and cadaverine are produced by pyridoxal 5′-phosphate-dependent L-ornithine, L-arginine, and L-lysine decarboxylases (ODC, ADC, LDC), respectively, from both the alanine racemase (AR) and aspartate aminotransferase (AAT) folds. Two homologous forms of AAT-fold decarboxylase are present in bacteria: an ancestral form and a derived, acid-inducible extended form containing an N-terminal fusion to the receiver-like domain of a bacterial response regulator. Only ADC was known from the ancestral form and limited to the Firmicutes phylum, whereas extended forms of ADC, ODC, and LDC are present in Proteobacteria and Firmicutes. Here, we report the discovery of ancestral form ODC, LDC, and bifunctional O/LDC and extend the phylogenetic diversity of functionally characterized ancestral ADC, ODC, and LDC to include phyla Fusobacteria, Caldiserica, Nitrospirae, and Euryarchaeota. Using purified recombinant enzymes, we show that these ancestral forms have a nascent ability to decarboxylate kinetically less preferred amino acid substrates with low efficiency, and that product inhibition primarily affects preferred substrates. We also note a correlation between the presence of ancestral ODC and ornithine/arginine auxotrophy and link this with a known symbiotic dependence on exogenous ornithine produced by species using the arginine deiminase system. Finally, we show that ADC, ODC, and LDC activities emerged independently, in parallel, in the homologous AAT-fold ancestral and extended forms. The emergence of the same ODC, ADC, and LDC activities in the nonhomologous AR-fold suggests that polyamine biosynthesis may be inevitable.

The polyamine spermidine is found throughout bacteria, archaea, and eukaryotes (1), and phylogenetic evidence indicates that it was synthesized in the Last Universal Common Ancestor (LUCA) (2). At physiological pH, the three amines of spermidine are protonated, and consequently spermidine is positively charged and able to interact with negatively charged macromolecules (3). In *Escherichia coli*, 90% of spermidine is noncovalently bound to RNA, with only 3.8% in the unbound form (4). Spermidine is strongly associated with ribosome function and translation, and in eukaryotes and most archaea, the aminobutyl moiety of spermidine is transferred to a single lysine residue in eIF5A to eventually form the essential hypusine modification (5). Hypusinated eIF5A is required for translation of mRNAs encoding polyproline tracts, but it is also required for normal translation elongation and termination (6). Furthermore, in many eukaryotes and archaea, spermidine is also required for biosynthesis of long-chain polyamines such as spermine and thermospermine and branched chain polyamines (7). The function of spermidine in bacterial growth ranges from being essential, to being required for normal growth, to being dispensable for normal growth (8).

Diamine putrescine (1,4-diaminobutane) is the biosynthetic precursor of spermidine (and homospermidine). Spermidine is synthesized from putrescine by *S*-adenosylmethionine decarboxylase and spermidine synthase or by carboxyspermidine dehydrogenase and carboxyspermidine decarboxylase (7). The primordial evolutionary history of spermidine biosynthesis and its universal distribution in the domains of life has been accompanied by a profound diversification of its biosynthetic pathways, especially of putrescine (7). Putrescine can be synthesized indirectly from L-arginine by arginine decarboxylase (ADC) (Fig. 1*A*) or directly from L-ornithine by ornithine decarboxylase (ODC). The product of arginine decarboxylation, agmatine, can be converted to putrescine directly by agmatine ureohydrolase (agmatinase) or by the combined actions of agmatine deiminase (AIH) and *N*-carbamoylputrescine amidohydrolase (NCPAH). Nonhomologous convergent evolution, *i.e.*, the convergent evolution of the same enzymatic function from different protein folds, also known as nonhomologous isofunctional enzymes (9), has resulted in emergence of ADC from at least four different protein folds (10). Two forms of ADC are pyridoxal 5′-phosphate (PLP)-dependent enzymes, from the aspartate aminotransferase fold (AAT-fold), and the alanine racemase fold (AR-fold), and two are pyruvoyl cofactor enzymes. ODC is currently known to have evolved from only the PLP-dependent AAT- and AR-folds. Cadaverine (1,5-diaminopentane) is a diamine found primarily in bacteria and some plants and is produced from L-lysine by L-lysine decarboxylase (LDC) (Fig. 1*A*). In *E. coli*, cadaverine induces closure of outer membrane porins (11) and in the diderm firmicute *Selenomonas ruminantium* is required for anchoring peptidoglycan to the outer membrane (12). LDC evolved from the AAT-fold (13) and the AR-fold (14, 15) and from within another distinct origin within the AAT-fold (structurally similar to L-glutamate and DOPA decarboxylases) in an example of distantly homologous convergent evolution (16). In *E. coli*, AAT-fold ADC, ODC, and LDC can be found as acid-inducible forms that participate in acid resistance (17), involving corresponding dedicated antiporters that take up L-arginine, L-ornithine or L-lysine and export the decarboxylation products agmatine, putrescine, or cadaverine (Fig. 1*A*). The activity of the acid-inducible ADC, ODC, and LDC enzymes was first shown in *E. coli* cultures, then known as *Bacterium coli*, over 80 years ago (18). Paralogous AAT-fold ODC and LDC versions are present in *E. coli* that are constitutively expressed at normal physiological pH and are biosynthetic (19, 20).

**Figure 1:**
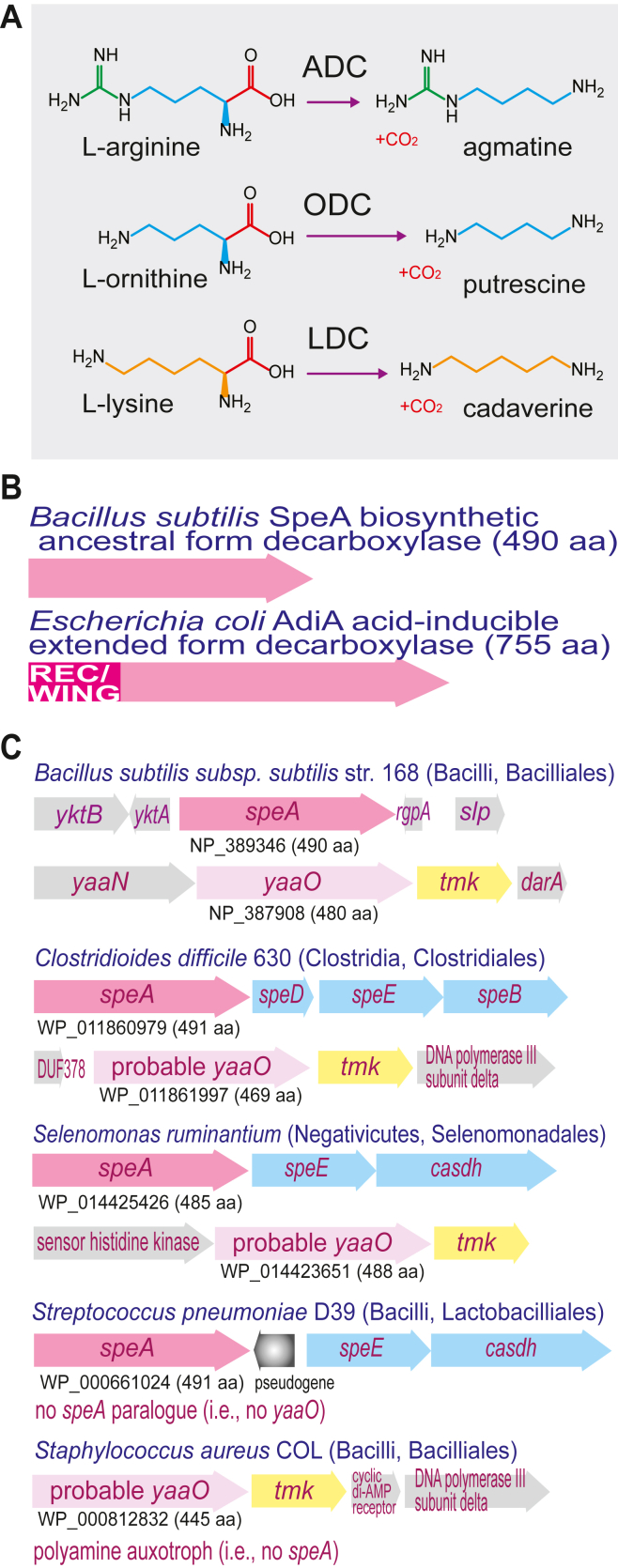
**L-arginine, L-ornithine, and L-lysine decarboxylation reactions and gene clusters of AAT-fold decarboxylase homologues.***A*, substrates and products of L-arginine, L-ornithine, and L-lysine decarboxylases (ADC, ODC, LDC). *B*, ancestral form and extended form of AAT-fold arginine decarboxylases. *C*, gene clusters from firmicute bacteria encoding functionally characterized arginine decarboxylases (SpeA/ADC), or SpeA paralogues (YaaO). ORFs encoding spermidine biosynthetic enzymes are shown in *blue*. REC, CheY-like response regulator receiver domain; *speA*, arginine decarboxylase; *tmk*, thymidylate kinase; *speB*, agmatine ureohydrolase/agmatinase; *speD*, *S*-adenosylmethionine decarboxylase; *speE*, spermidine synthase, *casdh*, carboxyspermidine dehydrogenase.

AAT-fold basic amino acid decarboxylases are found in two structural versions (Fig. 1*B*): a shorter (c. 480–490 amino acids) ancestral version represented by the ADC (SpeA) of *Bacillus subtilis* (21), and longer, “winged” versions such as the *E. coli* decarboxylases (usually c. 710–755 amino acids). The longer winged/extended (from here-on “extended”) versions are a fusion between an N-terminal CheY-like response regulator receiver domain (REC domain, c. 150 amino acids) and the ancestral form decarboxylase, fused to the C-terminus of the REC domain (22). Extended forms are derived from an ancestral form; whereas the extended forms exist as ADC, ODC, and LDC enzymes, until now only ADC has been identified in the ancestral form. To simplify enzyme descriptions, the ancestral forms will be referred to as aADC, aODC, and aLDC, and the longer extended/wing forms as exADC, exODC, and exLDC. All decarboxylases herein are from the AAT-fold unless otherwise stated. Extended-form ADC/ODC/LDC are found extensively in Proteobacteria and some Firmicutes, whereas aADC has been functionally identified only in the Firmicutes. The basic structure of the extended decarboxylases is a dimer of identical monomers, and the N-terminal REC domain is required for formation of a decameric form (a pentameric ring of dimers) in acid-inducible exADC (23) and exLDC (13, 24), in constitutive exLDC (25, 26, 27, 28), and a dodecameric form in *Lactobacillus* exODC (29). The functionally characterized aADCs from firmicute species are found in *B. subtilis* (21, 22), *S. ruminantium* (30), *Clostridioides difficile* (22), and *Streptococcus pneumoniae* (31). Given the evolution of multiple routes for polyamine biosynthesis, we sought to determine whether the ancestral form decarboxylases are present only as ADC, or whether ODC and LDC ancestral enzymes also exist.

## Results

### AAT-fold ancestral decarboxylase homologues unrelated to polyamine biosynthesis

When attempting to determine whether ancestral forms of ODC or LDC are encoded in bacterial genomes, several confounding factors must be considered. There is, for example, a paralogous copy (*yaaO*) of the aADC-encoding gene (*speA*) found in *B. subtilis*, with the two encoded protein sequences exhibiting 36% identity (Fig. 1*C*). While deletion of *speA* in *B. subtilis* abolishes polyamine (spermidine) accumulation, deletion of *yaaO* has no effect on polyamine content (21, 22). Recently, it has been established in *B. subtilis* that *yaaO* is involved in influencing the level of 5-aminopentanoylation of translation factor EF-P, and this 5-aminopentanol modification is likely to be found only in firmicute species (32). TBLASTN analysis indicates that *speA*/*yaaO* paralogous gene pairs are found throughout the Firmicutes phylum. In *B. subtilis*, *yaaO* is found immediately upstream of thymidylate kinase (*tmk*), and one of the two *speA*/*yaaO* gene pairs in other firmicute species is also found immediately upstream of a *tmk* homologue (Fig. 1*C*). Although *Staphylococcus aureus* is a polyamine auxotroph, lacking any polyamine when grown in chemically defined medium (33, 34), it does encode a putative *yaaO* homologue immediately upstream of *tmk* (Fig. 1*C*).

Homologues of ancestral *speA* are also found extensively in genomes of Cyanobacteria and Actinobacteria as a single genomic copy. In both these phyla, prior evidence suggests that the *speA* homologues are not involved in polyamine biosynthesis and are unlikely to encode aADC, aODC, or aLDC. In the nitrogen-fixing filamentous cyanobacterium *Anabaena* sp. PCC 7120, deletion of the AR-fold ADC abolished agmatine and homospermidine biosynthesis, even though a homologue of AAT-fold SpeA (WP_010999013; 40% protein identity to *B. subtilis* SpeA) is encoded in the genome (35). Similarly, deletion of the two AR-fold *speA* homologues of the unicellular cyanobacterium *Synechocystis* sp. PCC 6803 essentially eliminated spermidine production even though an ancestral form AAT-fold SpeA homologue (WP_010871419; 40% protein identity to *B. subtilis* SpeA) is encoded in the genome (36). Actinobacterium *Mycolicibacterium smegmatis*, a model for the TB agent *Mycobacterium tuberculosis*, is a polyamine auxotroph containing no detectable polyamines (37) but encodes an ancestral form AAT-fold SpeA homologue (WP_011726793; 34% protein identity to *B. subtilis* SpeA). The function of these cyanobacterial and actinobacterial SpeA homologues is unknown. Thus, of the many thousands of *B. subtilis* SpeA homologues encoded in bacterial genomes, a considerable number are unlikely to be aADC, aODC, or aLDC.

### Identification of ancestral form ornithine and bifunctional ornithine/lysine decarboxylases

**Figure 2:**
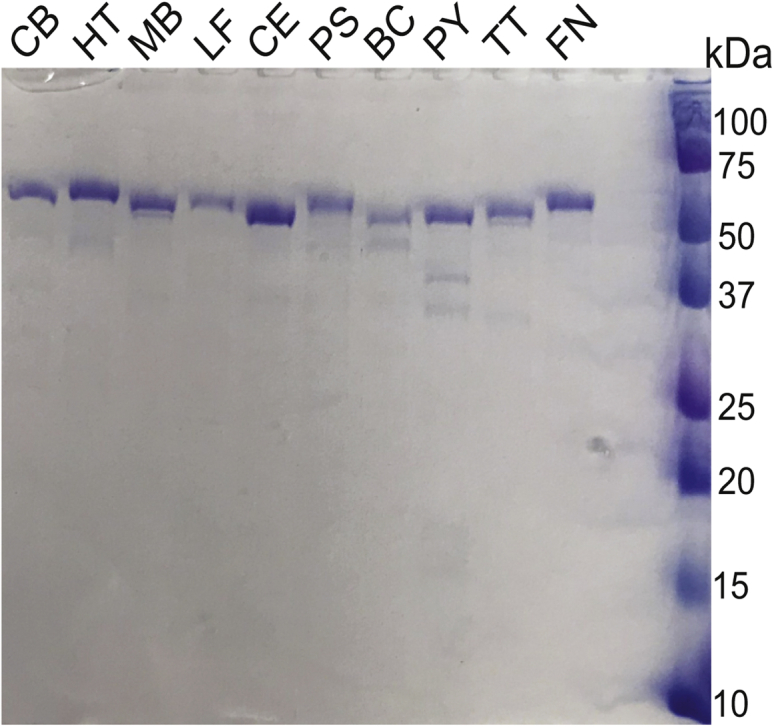
**Purified ancestral form AAT-fold decarboxylase recombinant proteins used in this study.** After expression of relevant ORFs from pET28 in *E. coli* BL21, purified recombinant proteins were separated by SDS-PAGE and stained with Coomassie dye. CB, *Clostridium botulinum* F str. Langeland (WP_012100824, 486 aa, 53.35 kDa); HT, *Hungateiclostridium thermocellum* (WP_011838304, 486 aa, 53.33 kDa); MB, *Methanomicrococcus blatticola* (WP_133517158, 483 aa, 53.48 kDa); LF, *Leptospirillum ferrooxidans* (WP014449684, 487 aa, 53.43 kDa); CE, *Caldisericum exile* (WP_014453687, 482 aa, 53.83 kDa); PS, *Peribacillus simplex* (WP_096338298, 485 aa, 53.01 kDa); BC, *Bacillus cihuensis* (WP_028393003, 485 aa, 53.06 kDa); PY, *Psychrilyobacter* sp. S5 (WP_114642662, 480 aa, 53.71 kDa); TT, *Thermoanaerobacterium thermosaccharolyticum* (WP_013298087, 495 aa, 54.74 kDa); FN, *Fusobacterium necrophorum* (WP_005954404, 490 aa, 54.41 kDa).

We noted an indication for a possibly active aODC from a report published over 50 years ago (38) that *Clostridium botulinum* 62-A was able to ferment ^14^C-labeled L-ornithine to NH_3_, CO_2_, acetate, propionate, valerate, and butyrate, but a portion of the L-ornithine was also decarboxylated to putrescine. Accordingly, we determined from PBLAST searches that *C. botulinum* F str. Langeland does not encode an extended form of SpeA/SpeC (*i.e.*, exADC/exODC), nor does it encode an AR-fold ADC or ODC. It does, however, encode a paralogous pair of ancestral SpeA homologues (WP_012100824, 486 aa; WP_011987181, 482 aa). The WP_011987181 ORF is immediately upstream of *tmk* and is therefore likely to be a YaaO orthologue. However, WP_012100824 was potentially an aADC rather than an aODC because the genome also encodes homologues of AIH (WP_012100824; 410 aa) and NCPAH (WP_003405206; 278 aa), which would suggest a full pathway from arginine to putrescine. We reasoned that in the light of the report of some L-ornithine decarboxylating activity in *C. botulinum*, the WP_012100824 AAT-fold decarboxylase homologue could either be a bifunctional ADC/ODC or a specific ODC, with AIH/NCPAH being involved in catabolism of externally derived agmatine. KEGG database (39) interrogation indicated that *C. botulinum* F str. Langeland is an arginine and ornithine auxotroph, and so the ADC/ODC substrates would have to be acquired externally. The potential *speA* homologous ORF from *C. botulinum* F str. Langeland was synthesized, expressed in *E. coli* BL21, and then purified (Fig. 2). After purification, the protein decarboxylase activity was assayed by detection of CO_2_ release at 27 °C, pH7.7 with L-arginine, L-ornithine, or L-lysine as substrates. The enzyme was found to be specific for L-ornithine, with a *k*_cat_/*K*_m_ for ornithine 313-fold greater than with L-arginine and 138-fold greater than with L-lysine (Table 1). Substrate preference of the *C. botulinum* enzyme for L-ornithine was manifested by a lower *K*_m_ and higher turnover rate than with L-arginine or L-lysine.

**Table 1:** Kinetic constants determined for ancestral form AAT-fold decarboxylases from *Clostridium botulinum*, *Thermanerobacterium thermosaccharolyticum*, *Fusobacterium necrophorum*, and *Hungateiclostridium thermocellum*

Species/enzyme	Substrate	*K*_m_ (mM)	*k*_cat_ (s^−1^)	*k*_cat_/*K*_m_ (M^−1^s^−1^)
*C. botulinum* ODC	L-ornithine	0.60 ± 0.030	2.8 ± 0.14	4700 ± 15
*C. botulinum* ODC	L-arginine	3.8 ± 0.25	0.56 ± 0.020	15 ± 5.8
*C. botulinum* ODC	L-lysine	2.0 ± 0.090	0.67 ± 0.030	34 ± 11
*T. thermosaccharolyticum* O/LDC	L-ornithine	0.70 ± 0.060	0.87 ± 0.090	1300 ± 11
*T. thermosaccharolyticum* O/LDC	L-arginine	0.66 ± 0.10	0.38 ± 0.060	580 ± 15
*T. thermosaccharolyticum* O/LDC	L-lysine	0.50 ± 0.010	0.57 ± 0.010	1100 ± 9.8
*F. necrophorum* ODC	L-ornithine	0.65 ± 0.030	1.1 ± 0.050	1700 ± 31
*F. necrophorum* ODC	L-arginine	19 ± 2.3	0.10 ± 0.010	5.5 ± 0.23
*F. necrophorum* ODC	L-lysine	4.0 ± 1.7	0.11 ± 0.040	27 ± 0.34
*H. thermocellum* ADC	L-ornithine	ND	ND	ND
*H. thermocellum* ADC	L-arginine	0.13 ± 0.010	0.050 ± 0.0010	410 ± 11
*H. thermocellum* ADC	L-lysine	ND	ND	ND

All assays performed in triplicate at 26 °C (±SD).

Abbreviation: ND, no detectable activity.

The closest homologues of the *C. botulinum* aODC identified by BLASTP are found in other members of the Clostridiales order, including *C. tetani* (WP_115605872; 486 aa), the causative agent of tetanus, and *C. perfringens* (WP_142418848; 487 aa), a food-borne pathogen and cause of gas gangrene. We noted that relatively close homologues are also found in the Thermanerobacterales order of Clostridia and decided to examine the substrate preference of the encoded WP_013298087 (495 aa) protein from *Thermoanaerobacterium thermosaccharolyticum* (67% protein identity with the *C. botulinum* aODC), a biotechnologically important organism used for the conversion of cellulosic biomass into useful chemicals (40). To our surprise, the purified recombinant enzyme (Fig. 2) from *T. thermosaccharolyticum* exhibited an equal preference for L-ornithine and L-lysine, with 2.2-fold less preference for L-arginine (Table 1). The *T. thermosaccharolyticum* enzyme is approximately 3.7-fold less efficient with L-ornithine compared to the *C. botulinum* ODC, primarily due to a lower turnover rate. A paralogous protein (WP_013296644; 473 aa) is encoded in the *T. thermosaccharolyticum* genome, but this ORF is immediately upstream of *tmk* and therefore probably encodes a YaaO orthologue.

We also noticed that many *Fusobacterium* species (phylum Fusobacteria) encode single gene homologues of the *C. botulinum* aODC, and we examined the substrate preference of the purified encoded WP_005954404 (490 aa) recombinant protein (Fig. 2) of *Fusobacterium necrophorum*, a cause of Lemierre's syndrome and pharyngitis (41). Compared with the *C. botulinum* aODC, the *F. necrophorum* enzyme is even more specific for L-ornithine, exhibiting a 63-fold preference for L-ornithine over L-lysine and a 309-fold preference over L-arginine (Table 1). As a comparator enzyme, we chose to examine the ancestral form *speA* homologue from the Clostridiales species *Hungateiclostridium thermocellum* (also known as *Clostridium thermocellum*, *Acetivibrio thermocellus*). The spermidine synthase gene from this species was shown recently to be essential for normal planktonic growth (42), in contrast to the *speA* gene of *B. subtilis*, which is dispensable for normal planktonic growth (22). The likely origin of putrescine biosynthesis in *H. thermocellum* is the ancestral form decarboxylase WP_011838304 (486 aa), which exhibits only 50% protein identity with the *C. botulinum* ODC. Kinetic analysis of the *H. thermocellum* purified recombinant enzyme (Fig. 2) revealed activity with L-arginine but no detectable or barely detectable activity with L-ornithine and L-lysine (Table 1). The catalytic efficiency of the *H. thermocellum* aADC activity was relatively low compared with the ODC activity of the aODC enzymes but is comparable to the *B. subtilis* SpeA aADC (22).

### Intrinsic substrate flexibility of the ancestral ODC, O/LDC, and ADC enzymes

Our characterized examples of ancestral form decarboxylases are either specific for L-ornithine or L-arginine or exhibit equal preference for L-ornithine and L-lysine. The relatively close sequence similarity of these homologous proteins suggested an inherent substrate flexibility of the last common ancestor progenitor enzyme that must have evolved into more specific substrate preferences. To explore the existing innate substrate flexibility of each enzyme, we used supraphysiological concentrations of each substrate (5 mM) and enzyme in *in vitro* reactions and detected the benzoylated polyamine product by LC-MS. Extracted Ion Chromatograms for dibenzoylated putrescine and cadaverine and tribenzoylated agmatine were used in product detection. Product formation was then compared with reactions using 10-fold less enzyme and substrate, including a shorter incubation time (30 min *versus* 5 min). Figure S1 shows that at high substrate/enzyme concentration in a 30 min reaction, each enzyme from *C. botulinum*, *T. thermosaccharolyticum*, *F. necrophorum*, and *H. thermocellum* decarboxylated all three substrates (L-ornithine, L-arginine, and L-lysine) to produce putrescine, agmatine, and cadaverine, respectively. However, Figure S1 shows that the preferred substrates identified by kinetic analysis produced the least product in these high substrate/enzyme conditions. For the *C. botulinum* aODC, agmatine was the most abundant product followed by cadaverine and then putrescine. The same pattern was observed with the *F. necrophorum* aODC, albeit agmatine was produced at a lower level compared with the *C. botulinum* ODC. With the *T. thermosaccharolyticum* aO/LDC, a very similar product accumulation pattern to the *C. botulinum* aODC was observed. In contrast, with the *H. thermocellum* aADC, cadaverine was the most abundant product, with lower levels for putrescine and agmatine. When 10-fold less substrate and enzyme and a shorter incubation were used, the product accumulation pattern reflected the substrate preference determined by kinetic analysis. The *C. botulinum* aODC accumulated primarily putrescine, then cadaverine, and greatly reduced agmatine; *F. necrophorum* aODC produced even less cadaverine and agmatine compared with putrescine; *T. thermosaccharolyticum* aO/LDC produced primarily putrescine and then cadaverine with greatly reduced agmatine; *H. thermocellum* aADC produced no detectable putrescine or cadaverine but did produce agmatine. These results illuminate inherent substrate flexibility and show that each enzyme is subject to strong product inhibition resulting from the kinetically preferred substrate.

### The Bacilli class of Firmicutes may encode only ADC activity

The ancestral form ODC enzymes that we identified in the Firmicutes phylum were found in the Clostridia class, and we wondered whether aODCs could be found within the large Bacilli class. Using BLASTP, we searched Bacilli proteins for those that were most diverged from the *B. subtilis* SpeA (aADC) and closest to the *C. botulinum* and *F. necrophorum* aODCs. We noticed that two of these proteins, homologues from *Peribacillus simplex* (WP_096338298; 485 aa) (also known as *Bacillus simplex*) (43) and *Bacillus cihuensis* (WP_028393003; 485 aa) (44), were encoded by ORFs adjacent to a divergently transcribed nitronate monooxygenase ORF. Notably, this was also the case for the *F. necrophorum* aODC ORF (Fig. S2), suggesting that the *P. simplex* and *B. cihuensis* proteins might exhibit ODC activity. The *B. cihuensis* and *P. simplex* genomes also encode paralogous proteins (WP_0283900542; 474 aa, and WP_096341007; 484 aa, respectively), located immediately upstream of *tmk*, indicating that these are YaaO orthologues. Purified recombinant *P. simplex* WP_096338298 and *B. cihuensis* WP_028393003 proteins (Fig. 2) were assayed for decarboxylation activity with L-ornithine, L-arginine, and L-lysine (Table 2). Against expectations, the *P. simplex* enzyme exhibited no activity toward L-ornithine or L-lysine and its *k*_cat_/*K*_m_ with L-arginine was 4.8 × 10^2^ M^−1^s^−1^. Similarly, the *B. cihuensis* enzyme produced no detectable activity with L-ornithine or L-lysine, but its *k*_cat_/*K*_m_ for L-arginine was 2.4 × 10^3^ M^−1^s^−1^.

**Table 2:** Kinetic constants determined for ancestral form AAT-fold decarboxylases from *Peribacillus simplex*, *Bacillus cihuensis*, *Psychrilyobacter* sp. S5, and *Methanimicrococcus blatticola*

Species/enzyme	Substrate	*K*_m_ (mM)	*k*_cat_ (s^−1^)	*k*_cat_/*K*_m_ (M^−1^s^−1^)
*P. simplex* ADC	L-ornithine	ND	ND	ND
*P. simplex* ADC	L-arginine	1.2 ± 0.050	0.57 ± 0.010	480.01 ± 26.79
*P. simplex* ADC	L-lysine	ND	ND	ND
*B. cihuensis* ADC	L-ornithine	ND	ND	ND
*B. cihuensis* ADC	L-arginine	0.45 ± 0.010	1.1 ± 0.010	2400.97 ± 99
*B. cihuensis* ADC	L-lysine	ND	ND	ND
*Psychrilyobacter* sp. S5 ADC	L-ornithine	ND	ND	ND
*Psychrilyobacter* sp. S5 ADC	L-arginine	0.21 ± 0.020	0.30 ± 0.0070	1400 ± 92
*Psychrilyobacter* sp. S5 ADC	L-lysine	6.6 ± 1.3	0.060 ± 0.0040	9.5 ± 1.1
*M. blatticola* ADC	L-ornithine	ND	ND	ND
*M. blatticola* ADC	L-arginine	0.65 ± 0.060	0.36 ± 0.010	550 ± 40
*M. blatticola* ADC	L-lysine	ND	ND	ND

All assays performed in triplicate at 26 °C (±SD).

Abbreviation: ND, no detectable activity.

### Ancestral form arginine decarboxylases encoded in Fusobacteria and Euryarchaeota phyla

As no aADC had been found outside of the Firmicutes phylum, we sought to identify aADC enzymes in other phyla. We had noticed that the Fusobacteriaceae family that contained *F. necrophorum* also contained species encoding a homologue relatively diverged from the *F. necrophorum* aODC. Furthermore, some Fusobacteriaceae species such as *Psychrilyobacter* sp. S5 possess an ancestral AAT-fold decarboxylase-encoding ORF in a gene cluster with *S*-adenosylmethionine decarboxylase, spermidine synthase, and agmatinase homologues. We purified the recombinant *Psychrilyobacter* sp. S5 ancestral AAT-fold protein (WP_114642662; 480 aa) (Fig. 2), which exhibits only 45% amino acid identity with the *F. necrophorum* aODC, and assayed its decarboxylation activity with L-ornithine, L-arginine and L-lysine. No detectable activity with L-ornithine was observed, negligible activity with L-lysine, but there was robust activity with L-arginine (*k*_cat_/*K*_m_ = 1.4 × 10^3^ M^−1^s^−1^) (Table 2). Thus, even within the same family, Fusobacteria species can encode highly specific forms of either aODC or aADC.

Archaea, with only a few exceptions, must produce agmatine through arginine decarboxylation in order to modify tRNA^ile^ on the cytidine of the anticodon CAT to form the agmatidine modification that allows discrimination between isoleucine and methionine codons (45, 46). Therefore, ADC is an essential enzyme in most archaea, and only pyruvoyl-dependent ADCs have so far been characterized from Archaea (47, 48). Using various ancestral aADC and aODC amino acid sequences from bacteria with BLASTP searches of archaeal genomes, we identified a number of archaeal genomes encoding a single ancestral form AAT-fold decarboxylase homologue. We screened those genomes, for example, where pyruvoyl-dependent ADC or AR-fold ADCs were absent, and selected one ORF (WP_133517158; 483aa) from *Methanomicrococcus blatticola*, a methanol- and methylamine-reducing methanogen (49) from the Methanomicrobia class of the Euryarchaeota phylum, for further analysis. The purified recombinant enzyme (Fig. 2) exhibited no detectable activity with L-ornithine or L-lysine, but the *k*_cat_/*K*_m_ with arginine was 5.5 × 10^2^ M^−1^s^−1^ (Table 2). This is the first functionally verified report of a PLP-dependent ADC from archaea, and the archaeal aADC possesses only 39% amino acid identity with the *B. subtilis* SpeA aADC.

### Identification of ancestral form ODC and LDC in Caldiserica and Nitrospirae phyla

To identify potential aODCs in more diverse phyla, we used TBLASTN to screen genomes for those that lack agmatinase and AIH but encode a single-copy ancestral AAT-fold decarboxylase homologue. Our reasoning was that if agmatine could not be converted to putrescine, the decarboxylase homologue was more likely to be an aODC than aADC. One candidate protein, which exhibited only 42% amino acid sequence identity with the *C. botulinum* aODC, was encoded in the genome of *Caldisericum exile*, an anaerobic, thermophilic, filamentous, thiosulfate-reducing bacterium (50) of the Caldiserica phylum (WP_014453687; 482 aa). The purified recombinant protein (Fig. 2) exhibited negligible activity with L-arginine or L-lysine but was active on L-ornithine, with a *k*_cat_/*K*_m_ of 5.9 × 10^2^ M^−1^s^−1^ (Table 3) and thus represents another highly specific form of the ancestral AAT-fold ODC. A second candidate aODC was identified in the genome of *Leptospirillum ferrooxidans*, a strict chemolithotroph limited to metabolizing only ferrous iron and pyrites (51), from the Nitrospirae phylum (WP014449684; 487 aa). When the purified recombinant protein (Fig. 2) was assayed at 26 °C, it exhibited decarboxylase activity for L-lysine, a negligible activity for L-ornithine, and no detectable activity with L-arginine (Table 3). This highly specific L-lysine decarboxylase activity was an unexpected substrate specificity since the decarboxylase ORF is immediately downstream of *S*-adenosylmethionine decarboxylase (WP_014449682; 157 aa) and spermidine synthase (WP_041774920; 306 aa) ORFs. We reassayed the decarboxylase enzyme at 30 °C and 37 °C (Table 3), which confirmed the *L. ferrooxidans* enzyme as the first example of an ancestral form AAT-fold, highly specific LDC.

**Table 3:** Kinetic constants determined for ancestral form AAT-fold decarboxylases from *Caldisericum exile and Leptospirillum ferroxidans*

Species/enzyme/temperature	Substrate	*K*_m_ (mM)	*k*_cat_ (s^−1^)	*k*_cat_/*K*_m_ (M^−1^s^−1^)
*C. exile* ODC (26 °C)	L-ornithine	0.18 ± 0.010	0.11 ± 0.0050	590 ± 36
*C. exile* ODC (26 °C)	L-arginine	0.54 ± 0.040	0.040 ± 0.0020	82 ± 3.2
*C. exile* ODC (26 °C)	L-lysine	ND	ND	ND
*L. ferroxidans* LDC (26 °C)	L-ornithine	28 ± 7.2	0.17 ± 0.020	4.2 ± 0.45
*L. ferroxidans* LDC (26 °C)	L-arginine	ND	ND	ND
*L. ferroxidans* LDC (26 °C)	L-lysine	0.37 ± 0.070	0.25 ± 0.020	700 ± 68
*L. ferroxidans* LDC (30 °C)	L-ornithine	NT	NT	NT
*L. ferroxidans* LDC (30 °C)	L-arginine	NT	NT	NT
*L. ferroxidans* LDC (30 °C)	L-lysine	0.44 ± 0.030	0.41 ± 0.030	930 ± 31
*L. ferroxidans* LDC (37 °C)	L-ornithine	35 ± 5.8	0.32 ± 0.030	9.2 ± 0.66
*L. ferroxidans* LDC (37 °C)	L-arginine	ND	ND	ND
*L. ferroxidans* LDC (37 °C)	L-lysine	0.55 ± 0.070	0.58 ± 0.070	1100 ± 39

All assays performed in triplicate (±SD).

Abbreviations: ND, no detectable activity; NT, not tested.

### A link between presence of aODC, L-ornithine/L-arginine auxotrophy, and dependency on the arginine deiminase system

To gain general insight into why an aODC rather than aADC activity might evolve for polyamine biosynthesis, we assessed whether genomes encoding aODCs could synthesize L-arginine or L-ornithine. The linear and cyclic pathways of L-arginine biosynthesis from L-glutamate in bacteria proceed *via* an L-ornithine intermediate, and other pathways exist where L-ornithine can be produced from L-arginine (52). By interrogation of the KEGG database (39), we found that *C. botulinum* str. F Langeland, *F. necrophorum*, and *C. exile* that encode a specific aODC do not have complete pathways for either L-ornithine or L-arginine biosynthesis and are thus likely to be L-ornithine and L-arginine auxotrophs. It was shown previously that *C. botulinum* 62-A takes up and ferments L-arginine and L-ornithine (38). Although *T. thermosaccharolyticum*, encoding a bifunctional O/LDC, does not encode L-arginine/L-ornithine biosynthesis, it does encode a complete L-lysine biosynthetic pathway from L-aspartate. Similarly, *L. ferrooxidans*, which encodes a specific LDC, does not encode L-arginine/L-ornithine biosynthesis but does encode a complete L-lysine biosynthetic pathway. For the aODC-encoding species to produce putrescine by ODC activity, they would have to take up exogenous L-ornithine. As L-ornithine is not found in proteins, its environmental concentration is likely to be low. However, many species utilize the arginine deiminase system under anaerobic conditions to produce energy from conversion of exogenous L-arginine to L-ornithine, which is then excreted by the arginine/ornithine antiporter ArcD (53). The aODC-encoding species may be dependent upon coexistence with other species possessing an active arginine deiminase system for cross-feeding of L-ornithine. Consistent with this idea, growth of *Fusobacterium nucleatum* in a dual species biofilm model with *Streptococcus gordonii* was previously found to be dependent on the presence in *S. gordonii* of the *arcD*-encoded L-arginine/L-ornithine antiporter of the arginine deiminase system (54). Deletion of the *arcD* gene reduced accumulation of *F. nucleatum* in the dual species biofilm and decreased L-ornithine excretion from *S. gordonii*. Supplementation with L-ornithine restored accumulation of *F. nucleatum* in the dual species biofilm with *S. gordonii* ΔarcD.

**Figure 3:**
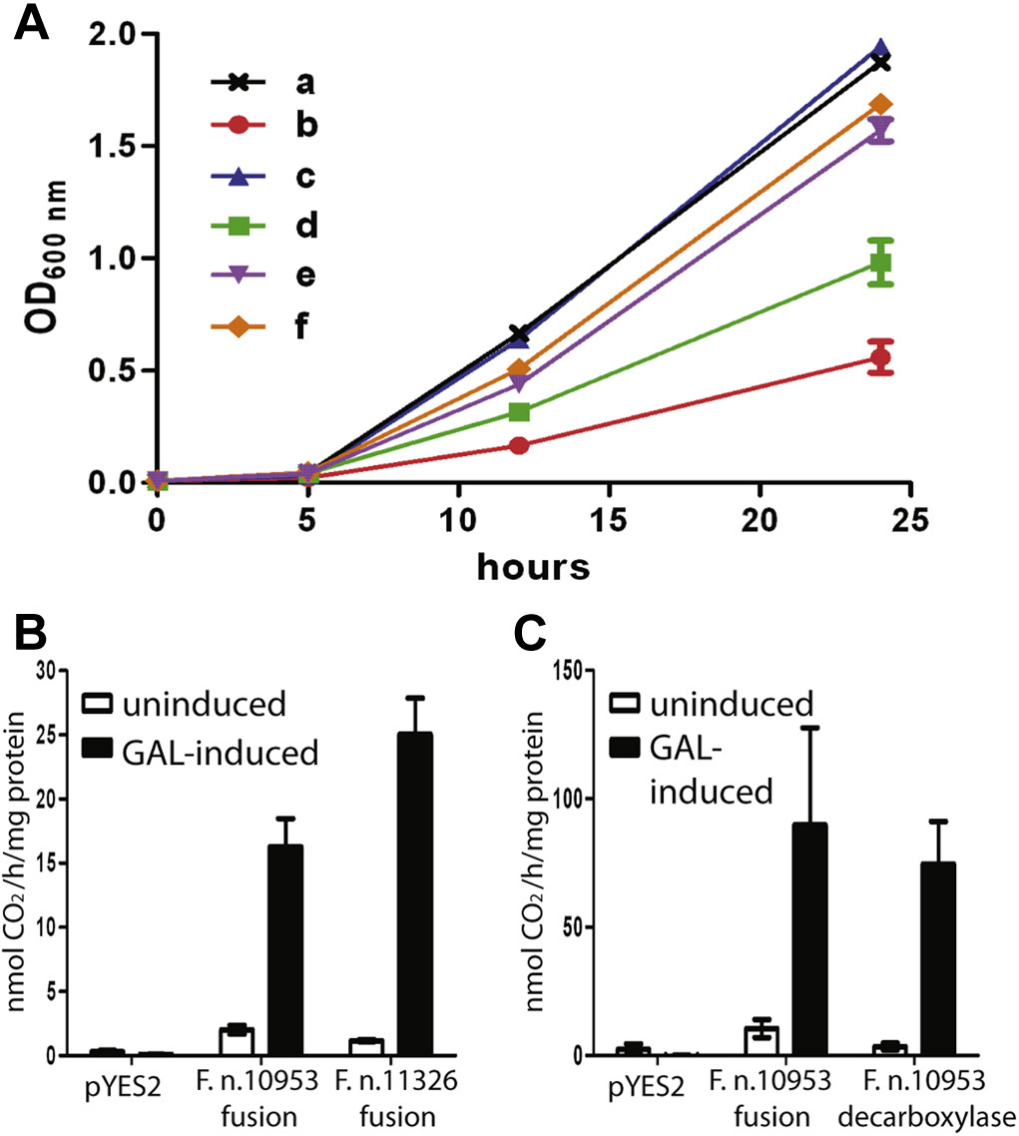
**Growth complementation of yeast ODC-deficient strain by *Fusobacterium nucleatum* ODC and corresponding ODC activity in cell extracts.***A*, Growth of transformed *S. cerevisiae* strains expressing the whole *F. nucleatum* fusion protein or the ODC-encoding portion only. Strains were grown in SD medium as described in experimental procedures. Data represent the means of triplicate cultures ±standard error. *a*, parental strain (BY4742); *b*, ΔSPE1 (ornithine decarboxylase gene deletion); *c*, ΔSPE1 + 1 mM putrescine; *d*, ΔSPE1 transformed with pYES2 empty vector; *e*, ΔSPE1 expressing *F. nucleatum* ATCC 10953 ODC-encoding portion; *f*, ΔSPE1 expressing *F. nucleatum* ATCC 10953 complete fusion protein. *B*, *In vitro* ODC activities determined with an L-[1-^14^C]ornithine assay in yeast cell extracts expressing *F. nucleatum* fusion proteins. *F. nucleatum* genes from strains ATCC 10953 and NCTC 11326 were transcribed from the GAL1 promoter, in the parental BY4742 strain and induced with galactose. *S. cerevisiae* BY4742 transformed with the empty pYES2 vector was used as a control. Data represent the means of six samples ±S.E. *C*, *In vitro* ODC activities in yeast cells expressing the whole *F. nucleatum* subsp. *polymorphum* ATCC 10953 fusion protein or only the ODC-encoding portion. *F. nucleatum* ORFs were transcribed from the GAL1 promoter, and induced with galactose. BY4742 transformed with the empty pYES2 vector was used as a control. Data represent the means of four samples ±S.E.

An ancestral form AAT-fold decarboxylase, highly homologous to the *F. necrophorum* ODC, is encoded in *F. nucleatum* (although it is fused to the N-terminus of an arginase/agmatinase homologue, and such fusions are found only in the Fusobacteriaceae), and abundance of this protein was increased in a dual biofilm with *S. gordonii* compared with *F. nucleatum* alone (55). To facilitate expression of this relatively large fusion protein, we expressed the *F. nucleatum* decarboxylase/arginase homologue fusion protein from two strains (*F. nucleatum* subsp. *polymorphum* ATCC 10953, and subsp. *fusiforme* NCTC 11326) in an ODC gene deletion strain (ΔSPE1) of *Saccharomyces cerevisiae* BY4742, as the complete fusion protein, and with just the decarboxylase domain (Fig. 3*A*). Expression of either the complete fusion protein from each strain (WP_005898385, 783 aa or WP_005913097, 783 aa; 759/783 protein identity) or only the decarboxylase domain (WP_005898385) restored growth to the *S. cerevisiae* ΔSPE1 gene deletion strain. Analysis of ODC activity using an L-[1-^14^C]ornithine assay from cell extracts of the BY4742 parental strain transformed with the same plasmids revealed L-ornithine decarboxylation activity with the complete fusion proteins (Fig. 3*B*) and the isolated decarboxylase domain (Fig. 3*C*). The ODC specific activity of the parental yeast strain expressing the whole fusion proteins was more than 40-fold above the native yeast endogenous activity, and the isolated decarboxylase domain exhibited similar activity to the whole fusion protein, confirming the fusion protein as an aODC. The arginase/agmatinase domain is unlikely to be active as two critical aspartate residues required for manganese ion coordination have been replaced by serine and asparagine (Fig. S3). A similar example of an inactive arginase has been characterized previously in the parasite *Trypanosoma brucei* (56). Although the evidence is correlative, it is suggestive that in some species aODC evolved to compensate for L-ornithine/L-arginine auxotrophy when easily obtainable L-ornithine was present in the environment due to other community species utilizing the arginine deiminase system.

### Evolutionary diversification of the AAT-fold basic amino acid decarboxylase homologues

**Figure 4:**
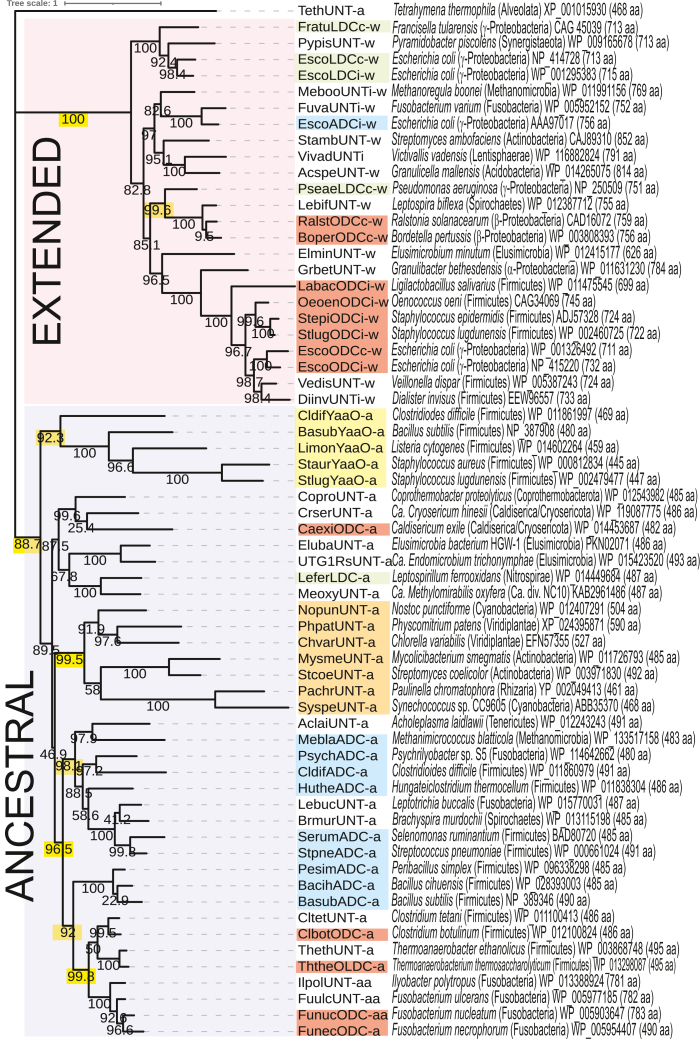
**Phylogeny of AAT-fold decarboxylase homologues.** A maximum likelihood tree was constructed using IQTREE based on an alignment of amino acid sequences made by MUSCLE, and 1000 ultrafast bootstrap results are shown as % values on the tree. Species names are presented with relevant phyla in parentheses, followed by GenBank protein accession number and the corresponding full protein size in amino acids. Protein names include ODC (L-ODC), ADC (L-ADC), LDC (L-LDC), OLDC, (bifunctional L-O/LDC), UNT (untested, *i.e.*, unknown function), i- (inducible), c- (constitutive), -w (contains N-terminal response regulator receiver (REC) domain homologous sequence, or “wing”), -a (ancestral, without N-terminal REC domain), -aa (ancestral decarboxylase with C-terminal arginase-like domain). The N-terminal and C-terminal regions of each amino acid sequence were trimmed to aid the alignment (eg., the trimmed *B. subtilis* str. 168 sequence starts at amino acid position seven and extends to position 483 of the 490 amino acid ORF). Accordingly, N-terminal REC domains and C-terminal arginase-like domains were removed from the relevant sequences. Color blocks represent similar enzymatic activities. Confirmation of enzymatic activity and gene identity: FratuLDCc-w (28), EscoLDCc-w (20, 72), EscoLDCi-w (73), PseaeLDCc-w (26), RalstODCc-w (74), BoperODCc-w (75), LabacODCi-w (76), OeoenODCi-w (77), StepiODCi-w (78), StlugODCi-w (79), EscoODCc-w (80), EscoODCi-w (81), CldifADC-a (22), SerumADC-a (30), StpneADC-a (31), BasubADC-a (21). From the current study, CaexiODC-a, Lefer LDC-a, MeblaADC-a, PsychADC-a, HutheADC-a, PesimADC-a, BacihADC-a, ClbotODC-a, ThtheOLDC-a, FunucODC-aa, FunecODC-a. Bootstrap support for important nodes are highlighted in *yellow*.

Until our current study, the only known ancestral fold decarboxylases were ADC enzymes from the firmicute species *B. subtilis* (monoderm), *Selenomonas ruminatium* (diderm), *S. pneumoniae* (monoderm), and *C. difficile* (monoderm) (Fig. 1*C*). Our study has discovered ancestral form ODC, LDC, and bifunctional O/LDC, as well as additional ADC enzymes. We aligned diverse functionally characterized and uncharacterized ancestral form and extended form amino acid sequences after removing the N-terminal REC domains, C-terminal arginase-like domains where relevant, and trimming all sequences at the N- and C-termini to aid alignment. After aligning sequences with MUSCLE and producing an unrooted Maximum Likelihood phylogenetic tree using IQTREE, it is notable that all extended form sequences (*i.e.*, with an N-terminal REC domain) form a highly supported clade (100% bootstrap value) distinct from the ancestral form sequences (Fig. 4). In addition to sequence divergence, the extended form proteins, *i.e.*, ADC, ODC, and LDC all possess at least eight short stretches of amino acid insertions (totaling approximately at least 67 aa) in conserved positions in the decarboxylase domain relative to all ancestral form proteins (Fig. S4). The confirmed independent evolutionary history of ancestral form and the derived extended form decarboxylases means that L-ornithine, L-arginine, and L-lysine decarboxylases have emerged independently in each of the two homologous groups. One decarboxylase, although it is unclear whether it was ADC or ODC from the ancestral form, acquired the N-terminal REC domain and acquired the eight small insertions to give rise to the first extended forms. It is not clear whether the original activity was ADC or ODC, or whether the acquisition of the N-terminal REC domain or the amino acid insertions came first. However, it seems more plausible that the single fusion event preceded and possibly caused the multiple insertions, as discussed later.

Among the ancestral form sequences, distinct clades can be seen for the YaaO homologues (92.3% bootstrap support), and for a group of sequences (99.5% boostrap support) that are found in actinobacteria and cyanobacteria, and in eukaryotes with chloroplasts (Viridiplantae) or with cyanobacterial endosymbionts (*Paulinella* in Rhizaria). The aODC encoded by *C. exile* from the Caldiserica phylum may have emerged independently of the aODCs of the Firmicutes and Fusobacteria phyla. Consistent with this, the *C. exile* aODC amino acid sequence exhibits 39% and 42% identity with the aODCs of *F. necrophorum* and *C. botulinum* F str. Langeland, respectively, whereas, the *B. subtilis* SpeA aADC exhibits 47.6 and 48% identity with the aODCs of *F. necrophorum* and *C. botulinum* F str. Langeland, respectively. However, it is also possible that there is simply a greater degree of vertical divergence between the *C. exile* aODC and the firmicute and fusobacterial ODCs. Curiously, among the extended form decarboxylase sequences, the exLDC of *Pseudomonas aeruginosa* is located in a highly supported clade (99.6% bootstrap support) with the functionally characterized exODCs of *Bordetella pertussis* and *Ralstonia solanacearum* and not in the clade (100% bootstrap support) containing the exLDCs from *E. coli* and *Francisella tularensis*. The phylogenetic tree confirms previous findings (26) that the *E. coli* constitutive exODC and exLDC are recent gene duplications of the acid-inducible forms.

## Discussion

Until this current study, the only known catalytic activity in the ancestral form AAT-fold decarboxylase homologues was the decarboxylation of L-arginine. When we set out to determine whether ODC was also present among the homologues, we were cautious because not all homologues were likely to be ADC, ODC, or LDC enzymes. Indeed, the homologues found in the Cyanobacteria and Actinobacteria are probably not polyamine-related. Consistent with this conclusion, most polyamine-producing cyanobacteria encode an ADC from the AR-fold, that is the likely origin of the algal and plant ADC (57). There is currently no indication of the biochemical function of the cyanobacterial and actinobacterial ancestral form AAT-fold decarboxylase homologues. Firmicute species encode two ancestral form AAT-fold decarboxylase paralogues, and in *B. subtilis*, one is ADC and the other YaaO. Recently, it has been discovered that YaaO is involved in the posttranslational 5-aminopentanol modification of translation factor EF-P, although its enzymatic activity is not yet determined. Such nonpolyamine related functions are also found among the AR-fold decarboxylases. In the AR-fold decarboxylase family, polyamine-related activities are represented by ADC, L-ODC, O/LDC, D-ODC, carboxyspermidine decarboxylase, and carboxynorspermidine decarboxylase (7). The nonpolyamine activities (58) are *meso*-diaminopimelate decarboxylase, the last step in lysine biosynthesis, *N*-citrylornithine decarboxylase, *N*-citryl-2,3-diaminopropionic acid decarboxylase, *O*-citrylserine decarboxylase, which are involved in polycarboxylate siderophore biosynthesis, MccE decarboxylates a 3-amino-3-carboxypropyl modification of the peptidyl-nucleotide translation inhibitor microcin C, BtrK decarboxylates a glutamate residue in the aminoglycoside antibiotic butirosin. Furthermore, the AR-fold ADC exists as two forms, an ancestral form that is the same size as other decarboxylases in the AR-fold, and an extended form approximately 250 amino acids longer, containing a 4-helical bundle internal insertion and a C-terminal extension that converts the extended ADC into a tetramer, rather than the dimer form of the other AR-fold decarboxylases (22, 59). There is, therefore, a precedent in the AR-fold decarboxylase family for the nonpolyamine related ancestral form AAT-fold decarboxylase homologues.

The emergence of ancestral ADC, ODC, and LDC enzymes from a common ancestor in the AAT-fold may be explained in part by the inherent ability of ADC, ODC, and LDC enzymes to decarboxylate all three amino acid substrates when enzyme and substrate concentrations are in excess, *i.e.*, there is already nascent substrate flexibility. This likely applies to the extended form of the ADC, ODC, and LDC enzymes with N-terminal REC domains. Indeed, the SpeC exODC of *E. coli* is capable of decarboxylating L-ornithine and L-2,4-diaminobutyrate (60). Similarly, extended form ODCs from the firmicute species *Oenococcus oeni* and *Lactobacillus brevis* are also able to decarboxylate L-lysine and 2,4-diaminobutyrate but at orders of magnitude less efficiency than L-ornithine (61).

Unlike *E. coli*, which encodes biosynthetic forms of both ADC (AR-fold) and ODC, the bacterial species that encode the ancestral form AAT-fold ODC encode only aODC, and similarly the species encoding ADC encode only aADC. What might explain the preference for ODC or ADC? The human pathogen *S. pneumoniae* is an ornithine and arginine auxotroph, and exogenous arginine is an essential amino acid for growth (62). It encodes an aADC, and its niche in the human host is more likely to contain arginine than ornithine. The arginine deiminase pathway of *S. pneumoniae* does produce ornithine from exogenously derived arginine, but the ornithine is excreted by the ArcD arginine-ornithine antiporter (63). In contrast, *F. nucleatum*, which is also an ornithine and arginine auxotroph, encodes aODC rather than aADC. Unlike *S. pneumoniae*, *F. nucleatum* does not encode the arginine deiminase pathway. Instead, this human oral commensal takes up exogenous ornithine produced by the arginine deiminase pathway of *S. gordonii*. In the examples of *S. pneumoniae* and *F. nucleatum*, the selective pressure to evolve or acquire by horizontal gene transfer, either ADC or ODC activities to produce putrescine may have been driven by arginine/ornithine auxotrophies and the relative abundance of extracellular arginine and ornithine in the respective ecological niches. The new ancestral form ODCs discovered in our study are all from anaerobic species: *C. botulinum*, *T. thermosaccharolyticum*, *F. necrophorum*, *F. nucleatum*, and *C. exile*. However, it is unlikely that an anaerobic lifestyle *per se* selects for ODC activity, as the aADC encoded by *Psychrilyobacter* sp. S5, which is in the same Fusobacteriaceae family as *F. necrophorum* and *F. nucleatum*, is also from an anaerobic species.

The extended form AAT-fold ADC, ODC, and LDC possess an N-terminal REC domain derived from a CheY-like receiver domain of a response regulator fused to the N-terminus of an ancestral form homologue (22). Besides the N-terminal REC domain, all the extended form enzymes contain at least eight small insertions in conserved positions, in the domain homologous to the ancestral form. The N-terminal REC domain extension and internal insertions therefore must have occurred in the common ancestor of the extended form enzymes before diversification into ADC, ODC, and LDC activities. It is unlikely that the presence of internal insertions *per se* has contributed to substrate specificity, since each exADC, exODC, and exLDC protein contains them, however, change of residues within the insertions could participate in substrate selection. It seems more plausible that the N-terminal REC domain was acquired before the insertions because there is no clear reason why the insertions would have occurred without the new quaternary structural possibilities that the REC domain confers. The first extended form acid-inducible decarboxylase must have also acquired a corresponding basic amino acid/diamine antiporter that evolved in tandem with the decarboxylase substrate diversification because the different antiporters are also homologous.

Emergence of ADC, ODC, and LDC activities occurred independently in the ancestral and extended form enzymes. This parallel emergence of ADC, ODC, and LDC activities represents pseudoconvergent evolution, *i.e.*, convergent evolution within closely homologous proteins from the same fold (64). Whatever the identity of the original ancestral form enzyme, *i.e.*, ADC or ODC (or LDC), each extant ancestral form exhibits an inherent substrate flexibility and can decarboxylate the other basic amino acids, albeit at lower efficiency. The parallel, independent emergence of the different preferred substrate specificities among the homologous ancestral and extended forms poses an interesting question about whether the same solutions to specification of substrate preference have evolved. A more distant form of pseudoconvergent evolution has occurred for LDC activity in species such as *Streptomyces coelicolor*, where the DesA LDC, involved in siderophore biosynthesis (16), has emerged from the glutamate decarboxylase family within the AAT-fold but bears little sequence similarity to the ancestral and extended form LDCs discussed here. Our study has discovered ODC and LDC ancestral forms and extended the phylogenetic diversity of the enzymes to Fusobacteria, Nitrospirae, Caldiserica, and Euryarchaeota phyla. Our discovery of the parallel, homologous but independent evolution of ADC, ODC, and LDC enzymes offers an opportunity to study repeated independent emergence of the same enzyme activities from closely homologous proteins.

## Experimental procedures

### Protein expression and purification

DNA transformation of *E. coli* BL21 cells was performed with 50 μl of competent cells that were maintained on ice. Approximately 20 ng of plasmid DNA in 1 μl was added to the competent cells and gently mixed. The mixture was kept on ice for 30 min and heat shocked at 42 °C for 90 s. Five-hundred microliter of LB medium was added to the tube and then shaken for 90 min at 37 °C. Two-hundred microliter of the transformed cells was then spread on LB solid agar plates with corresponding antibiotics and incubated overnight at 37 °C. Cells containing the decarboxylase ORFs in pET28a-TEV were grown to mid-log phase at 37 °C with aeration before addition of 0.2 mM IPTG (isopropyl-β-d-thiogalactopyranoside), and then cells were cultured overnight at 16 °C. Cells were resuspended in 100 mM HEPES buffer (pH 8.0), 50 mM NaCl, 5 mM imidazole, 20 μM PLP, 0.02% Brij35 and lysed in a cell disruptor at 10,000 psi. Lysate was centrifuged for 60 min to remove unbroken cells, debris, and insoluble material. The supernatant was applied to a 5 ml Hi-Trap chelating HP (GE Healthcare) column equilibrated with NiSO_4_ and buffer A, and the 6xHis-tagged proteins were eluted from the column with a gradient of 0–50% buffer B over 20 column volumes. Buffer A: 100 mM HEPES buffer (pH 8.0), 50 mM NaCl, 5 mM imidazole, 20 μM PLP, 0.02% Brij35; buffer B: 100 mM HEPES buffer (pH 8.0), 50 mM NaCl, 1.0 M imidazole, 20 μM PLP, 0.02% Brij35. Proteins were desalted by dialysis against 100 mM HEPES buffer (pH 8.0), 50 mM NaCl, 20 μM PLP, 0.02% Brij35 at 4 °C overnight. Protein purity was assessed using sodium dodecyl sulfate–polyacrylamide gel electrophoresis, and protein concentration was determined using a Biotek Multi-Mode Microplate reader under protein OD_280_ wavelength read with the molecular weight and protein extinction coefficients program.

### Cloning and expression of *F. nucleatum* fusion proteins

The ORFs encoding aODC-arginase/agmatinase fusion proteins were amplified by PCR from genomic DNA of *F. nucleatum* subsp. *polymorphum* ATCC 10953 (WP_005898385; 783 aa), and subsp. *fusiforme* NCTC 11326 (WP_005913097; 783 aa). PCR reactions included 50 ng genomic DNA, 10 nmol dNTPs, 20 pmol each of primers Fuso5′[BamH1] (5′-GGATCCATGTCTAAATTAGACCAAAATAAG-3′) and Fuso3′[Xho1] (5′-CTCGAGTTAATAATCTGGGTTCATCATATA-3′), and 2.5 U ExTaq polymerase (Takara). Amplification was performed for 20 cycles with: denaturation at 95 °C for 30 s, primer annealing at 60 °C for 30 s, and extension at 72 °C for 60 s; a final extension step at 72 °C for 5 min. The aODC domain of the *F. nucleatum* sp. *polymorphum* ATCC 10953 fusion protein was amplified by PCR from genomic DNA using primers Fuso5′[BamH1] and FusoODCR (5′-AATTCTCGAGTTATTCAATTACATT TATAGTTTC-3′). PCR products were cloned into pGEM-T Easy (Promega) and verified by DNA sequencing. The *F. nucleatum* fusion protein ORFs and isolated decarboxylase domain were cloned into the yeast expression plasmid pYES2 (Invitrogen), using BamH1 and Xho1 restriction sites. Yeast expression plasmids were introduced into *S. cerevisiae* BY4742 (parental strain; *MATαhis3Δ1leu2Δ0lys2Δ0ura3Δ0*) and its ΔSPE1 gene deletion derivative (Open Biosystems). For ODC activity assays, transformed cells were grown in 100 ml SD medium at 30 °C for 16 h with shaking; cells were harvested by centrifugation, washed in 10 ml SD medium lacking glucose, and resuspended in 2 ml SD medium lacking glucose. One milliliter of cells was used to inoculate 100 ml SD medium, and 1 ml was used to inoculate 100 ml SD medium lacking glucose, with 2% galactose. Each culture was incubated at 30 °C for 5 h with shaking. Cells were then pelleted by centrifugation and stored at –20 °C prior to being used in enzyme activity assays. For growth assays, transformed strains were grown overnight in 10 ml SD medium, diluted to an initial OD_600_ of 0.01 in 100 ml of fresh SD medium, and incubated as described above. ODC activity was assayed by quantification of released ^14^CO_2_ from ^14^C-labelled ornithine, as described previously (65).

### Enzyme assays and kinetic analysis

All reactions were performed in 200 μl volumes containing 50 mM HEPES pH 7.7, 100 mM NaCl, 20 μM PLP and 1 mM DTT. To this was added 100 μl of CO_2_ detection solution from an Infinity Carbon Dioxide liquid stable reagent (Thermo Scientific). The reactions contained 0–10 mM amino acid substrate and enzyme. A Biotek plate reader was used to detect CO_2_ *via* the detection solution. Assays were performed at 26 °C unless otherwise stated and monitored at OD_340_ with 10 s/read until an end reading at 40 min. Enzyme concentration varied depending on enzyme activity and was chosen to ensure that the reaction remained within the linear range. Kinetic calculations were made on data fit to a linear model, and the slope of this fit was taken as the rate of the reaction to obtain mean velocity V (absorbance units (AU)/min) using the automatic function of the plate reader. Reaction rate was transformed from AU/min to mM NADH/min using an extinction coefficient of 6.349 AU/mM/cm for NADH (66) and an approximate path length of 0.58 cm for assays performed in 200 μl final volume in a Corning Costar flat bottom 96-well plate.

### Enzyme reactions for liquid chromatography–mass spectrometry analysis

Enzyme reactions were performed in 200 μl volumes containing 100 mM HEPES pH 8.0, 50 μM PLP, 1 mM DTT, 5.0 mM or 0.5 mM L-arginine, L-ornithine or L-lysine, and 10 μM or 1 μM enzyme for 30 min or 5 min. Reactions were incubated at 26 °C and stopped by addition of 60 μl of 40% trichloroacetic acid. To benzoylate the reaction products, the reaction was added to 1 ml of 2 M NaOH and 10 μl benzoyl chloride, followed by vigorous mixing for 2 min. Two milliliter of diethyl ether was added, vortexed for 2 min, and left at room temperature for 30 min. The upper layer of diethyl ether containing the polyamines was transferred to a new tube and kept in a chemical hood until fully evaporated.

### Liquid chromatography–mass spectrometry analysis

Benzoylated samples were analyzed as previously described (67). Briefly, LC-MS analysis was performed on an Agilent 1290 Infinity HPLC system using an Eclipse XDB-C18 column (4.6 × 150 mm, 5 μm; Agilent) that was coupled to an Agilent 6130 single quadrupole ESI mass spectrometer run in the positive mode with a scan range of 50–1100 m/z. Liquid chromatography was carried out at a flow rate of 0.5 ml/min at 20 °C with a 5 μl injection volume, using a gradient elution with aqueous acetonitrile containing 0.1% formic acid.

### Phylogenetic analysis

Proteins were aligned initially by ClustalW, and this alignment was used to determine how much N-terminal and C-terminal sequence to trim from proteins for further alignment. Trimmed protein sequence alignment was performed with MUSCLE (68) using the European Bioinformatics Institute server (https://www.ebi.ac.uk/Tools/msa/muscle/). Aligned sequences in a ClustalW output file (.clw) were used to generate a Maximum Likelihood phylogenetic tree by using IQTREE (69) (http://iqtree.cibiv.univie.ac.at) with default settings for a protein alignment and 1000 ultrafast bootstrap analysis (70). Ultrafast bootstrap values can only be interpreted as high confidence support for a clade if the value is 95% or above. The resulting (.treefile) file was uploaded to iTOL (71) to draw the tree (https://itol.embl.de/upload.cgi) and then exported to Illustrator for annotation as an (.eps) file.

## Data availability

Data used for the study are presented or cited in the article or the supporting information.

## Supporting information

This article contains supporting information.

## Conflict of interest

M. A. P. holds the Sam G. Winstead and F. Andrew Bell Distinguished Chair in Biochemistry. All other authors declare that they have no conflicts of interest with the contents of this article.
