# Supplementary material for: Discovery of ancestral L-ornithine and L-lysine decarboxylases reveals parallel, pseudoconvergent evolution of polyamine biosynthesis
Source: J Biol Chem. 2021 Sep 21;297(4):101219. doi: 10.1016/j.jbc.2021.101219 (PMC8503589; doi:10.1016/j.jbc.2021.101219)
Supplement: Figures S1–S4 [file mmc1.pdf]

Supplemental Information

Discovery of ancestral L-ornithine and L-lysine decarboxylases reveals parallel, pseudoconvergent evolution of polyamine biosynthesis

Bin Li[1], Jue Liang[1], Colin C. Hanfrey[2,3], Margaret A. Phillips[1], Anthony J. Michael[1*]

[1]Department of Biochemistry, UT Southwestern Medical Center, Dallas, Texas, USA

[2]Institute of Food Research, Norwich, UK

[3]Current address: County Upper School, Beeton's Way, Bury St Edmunds, UK

Corresponding author: Anthony J. Michael

[*]E-mail: anthony.michael@utsouthwestern.edu

Figure S1. LC-MS analysis of benzoylated reaction products from purified recombinant ODC, O/LDC and ADC enzymes. Extracted Ion Chromatograms (EIC) corresponding to the mass for dibenzoylated putrescine (EIC = 297:298), tribenzoylated agmatine (EIC = 443:444) and dibenzoylated cadaverine (EIC = 311:312) are shown. Reactions were carried out with either 10 µM of enzyme and 5.0 mM amino acid substrate for 30 min, or with 1.0 µM of enzyme and 0.5 mM amino acid substrate for 5 min.

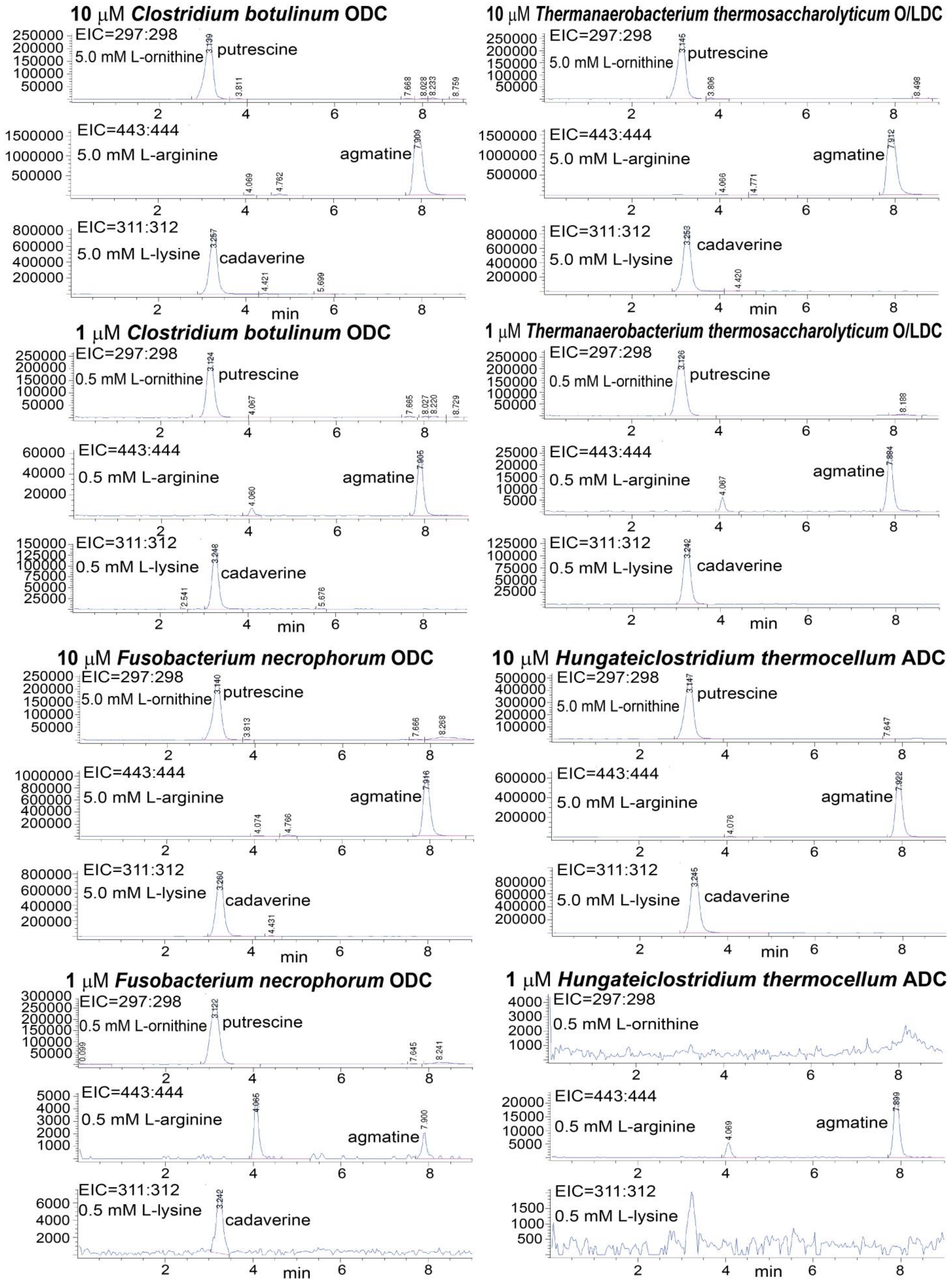
10 μM Clostridium botulinum ODC
EIC=297:298
5.0 mM L-ornithine
putrescine
EIC=443:444
5.0 mM L-arginine
agmatine
EIC=311:312
5.0 mM L-lysine
cadaverine
min
10 μM Thermanaerobacterium thermosaccharolyticum O/LDC
EIC=297:298
5.0 mM L-ornithine
putrescine
EIC=443:444
5.0 mM L-arginine
agmatine
EIC=311:312
5.0 mM L-lysine
cadaverine
min
1 μM Clostridium botulinum ODC
EIC=297:298
0.5 mM L-ornithine
putrescine
EIC=443:444
0.5 mM L-arginine
agmatine
EIC=311:312
0.5 mM L-lysine
cadaverine
min
1 μM Thermanaerobacterium thermosaccharolyticum O/LDC
EIC=297:298
0.5 mM L-ornithine
putrescine
EIC=443:444
0.5 mM L-arginine
agmatine
EIC=311:312
0.5 mM L-lysine
cadaverine
min
10 μM Fusobacterium necrophorum ODC
EIC=297:298
5.0 mM L-ornithine
putrescine
EIC=443:444
5.0 mM L-arginine
agmatine
EIC=311:312
5.0 mM L-lysine
cadaverine
min
10 μM Hungateiclostridium thermocellum ADC
EIC=297:298
5.0 mM L-ornithine
putrescine
EIC=443:444
5.0 mM L-arginine
agmatine
EIC=311:312
5.0 mM L-lysine
cadaverine
min
1 μM Fusobacterium necrophorum ODC
EIC=297:298
0.5 mM L-ornithine
putrescine
EIC=443:444
0.5 mM L-arginine
agmatine
EIC=311:312
0.5 mM L-lysine
cadaverine
min
1 μM Hungateiclostridium thermocellum ADC
EIC=297:298
0.5 mM L-ornithine
EIC=443:444
0.5 mM L-arginine
agmatine
EIC=311:312
0.5 mM L-lysine
min

Figure S2. Gene clusters containing nitronate monooxygenase and ancestral AAT-fold decarboxylase homologues.

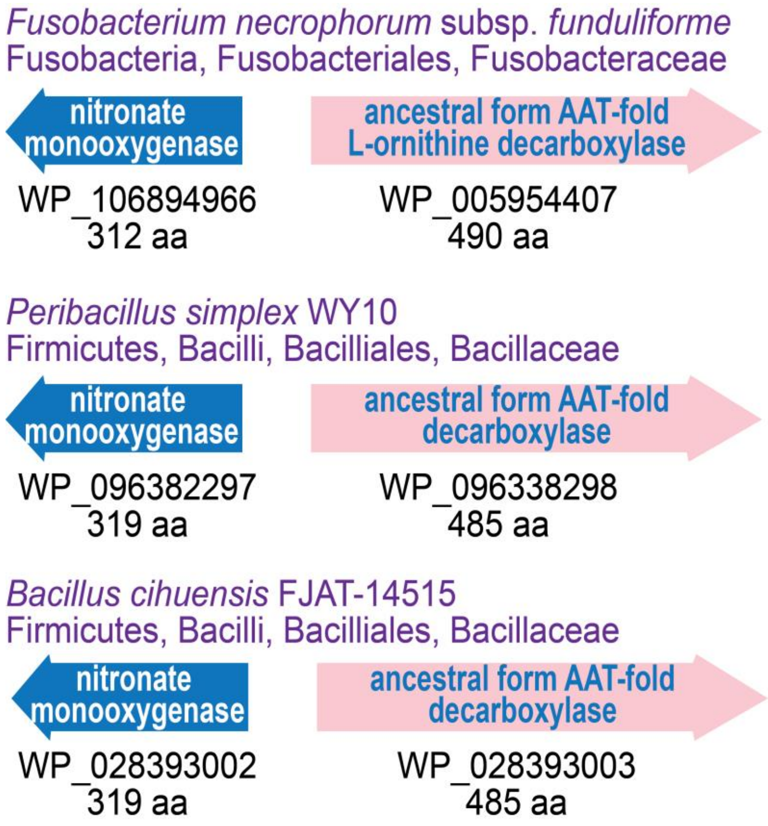

Supplemental Figure S3. Alignment of *Fusobacterium nucleatum* arginase domain with diverse arginase, agmatine ureohydrolase and aminopropylagmatine ureohydrolase sequences.

B.subAUH, *Bacillus subtilis* agmatine ureohydrolase [WP_003227545] (1); C.dif AUH, *Clostridioides difficile* CD196 agmatine ureohydrolase [CBA61606, PDB ID: 3LHL]; T.kod AAUH, *Thermococcus kodakarensis* KOD1 aminopropylagmatine ureohydrolase [WP_011249833] (2); T.the ARG, *Thermus thermophilus* HB27 arginase-like [AAS81474, PDB ID: 2EF4]; B.ant ARG, *Bacillus anthracis* str. A0248 arginase [WP_000711571] (3); F.nuc ARG, *Fusobacterium nucleatum* subsp. *nucleatum* ATCC arginase-like [AAL94697]; H.sap ARG1, human arginase-2 mitochondrial precursor [NP_001163, PDB ID: 1PQ3]; T.the AAUH, *Thermus thermophilus* HB8 aminopropylagmatine ureohydrolase [Q5SJ85] (4); D.rad AUH, *Deinococcus radiodurans* R1 agmatine ureohydrolase [WP_010889409, PDB ID: 1WOG] (5); E.col AUH, *Escherichia coli* str. K-12 substr. MG1655 agmatine ureohydrolase [NP_417412, PDB ID 7LOL]. The conserved residues required for binding the two manganese ions (5) are shown in red. The aberrant residues found in the *F. nucleatum* sequence that would normally be required for manganese binding, are indicated by the red triangles and highlighted in yellow.

1. Shimotohno, K. W., Hidaka, T., Morishita, T., and Endo, T. (2003) Molecular cloning of the gene for edeine B1 amidinohydrolase in addition to the agmatinase activity in Bacillus subtilis. *Biol. Pharm. Bull.* **26**, 262-265
2. Morimoto, N., Fukuda, W., Nakajima, N., Masuda, T., Terui, Y., Kanai, T., Oshima, T., Imanaka, T., and Fujiwara, S. (2010) Dual biosynthesis pathway for longer-chain polyamines in the hyperthermophilic archaeon Thermococcus kodakarensis. *J. Bacteriol.* **192**, 4991-5001
3. Viator, R. J., Rest, R. F., Hildebrandt, E., and McGee, D. J. (2008) Characterization of Bacillus anthracis arginase: effects of pH, temperature, and cell viability on metal preference. *BMC Biochem.* **9**, 15
4. Ohnuma, M., Terui, Y., Tamakoshi, M., Mitome, H., Niitsu, M., Samejima, K., Kawashima, E., and Oshima, T. (2005) N1-aminopropylagmatine, a new polyamine produced as a key intermediate in polyamine biosynthesis of an extreme thermophile, Thermus thermophilus. *J. Biol. Chem.* **280**, 30073-30082
5. Ahn, H. J., Kim, K. H., Lee, J., Ha, J. Y., Lee, H. H., Kim, D., Yoon, H. J., Kwon, A. R., and Suh, S. W. (2004) Crystal structure of agmatinase reveals structural conservation and inhibition mechanism of the ureohydrolase superfamily. *J. Biol. Chem.* **279**, 50505-50513


```
B.sub AUH   : -----MRFDEAYSGKVFIASRPEWE------EADAILYGMPMDWTVSYRPGSRFGPSRIREVSIG--LEEY-SPYLDRDLADLNFFDAGDIPLPFGNPQRSLDMIEEYVDSILEKG : 102
C.dif AUH   : -------------MNTFMSMDKNYE------ESNLIVFGVGFDGTTSNRPGARFASSSMRKEFYG--LETY-SPFLDLDLEDYNICDYGDLEISVGSTEQVLKEIYQETYKIVRDS :  94
T.kod AAUH  : -----MEFLYTYETLKLEFPLVEPE------KARFILLGVPFDGTTSYKAGARFGPTLIRQATLN--LESY-ILDYDLDIAELPIADIGDIAVVAGDPRKTADRVRETLEELKKAN : 102
T.the ARG   : --------------------------------ERVAVVGVPMDLGAN-RRGVDMGPSALRYARLLEQLEDLGYTVEDLGDVPVSLARASRRRGRGLAYLEEIRAAALVLKERLAAL :  83
B.ant ARG   : ------------------------M------KKEISVIGVPMDLGQM-RRGVDMGPSAIRYAGVIERIEEIGYDVKDMGDICIEREKEVDVN-TSLRNLTQVATVCNELASKVDHI :  84
F.nuc ARG   : -----------------DAIYLYTE------KMKNVLIGVQTNLGVN-KTGTEFGPDDL--------IQAYPDTFDEMELISVERQKE-DFNDKKLKFKNTVLDTCEKIAKRVNEA :  83
H.sap ARG1  : --------------------------------HSVAVIGAPFSQGQK-RKGVEHGPAAIREAGLMKRLSSLGCHLKDFGDLSFTPVPKDDLYNNLIVNPRSVGLANQELAEVVSRA :  83
T.the AAUH  : ------------MRLVFGEKDTPYE------EARVVVLPVPYDLSLSFLPGARRGPEAILLASRE--LEPF-LLELGAAPEEVGIHAAEPVPWVAGMAEESHRLIREEALRHLRAG :  95
D.rad AUH   : -MSGPAHLPYGGIPTFARAPLVQPDGD---WQADVAALGVPFDIALGFRPGARFAPRALREASLR-SVPPFTGLDGKTRLQGVTFADAGDVILPSLEPQLAHDRITEAARQVRGRC : 111
E.col AUH   : MSTLGHQYDNSLVSNAFGFLRLPMNFQPYDSDADWVITGVPFDMATSGRAGGRHGPAAIRQVSTNLAWEHNRFPWNFDMRERLNVVDCGDLVYAFGDAREMSEKLQAHAEKLLAAG : 116

B.sub AUH   : ----KFPMGMGGEHLVSWPVIKAMYKKYPDLAIIHFDAHTDLRVDYEG-EPLSHSTPIRKAAELIG-----------------PHNVYSFGIR--SGMKEEFEWAKENGMHISKFE : 194
C.dif AUH   : ----KVPFMIGGEHLVTLPAFKAVHEKYNDIYVIHFDAHTDLREEYNN-SKNSHATVIKRIWDIVG-----------------DNKIFQFGIR--SGTKEEFKFATEEKHTYMEIG : 186
T.kod AAUH  : P--KAIPILLGGEHSQTLGAVEALKPAS----YVVFDAHLDLRNSYED-NPYNHACVARRISELG-------------------VKEAIFGIR--SGTKEEVDFARERDIPWVHAR : 190
T.the ARG   : P-EGVFPIVLGGDHSLSMGSVAGAARGRR-VGVVWVDAHADFNTPETSPSGNVHGMPLAVLSGLGH---PRLTEVFRA---VDPKDVVLVGVR--SLDPGEKRLLKEAGVRVYTMH : 189
B.ant ARG   : IEEGRFPLVLGGDHSIAIGTLAGVAKHYKNLGVIWYDAHGDLNTEETSPSGNIHGMSLAASLGYGH---PTLVDLYGAYPKVKKENVVIIGAR--ALDEGEKDFIRNEGIKVFTMH : 195
F.nuc ARG   : VIDGYRPILVGGDHSISLGSVSGVS-LEKEIGVLWISAHGDMNTPESTLTGNIHGMPLALLQGLGD---RELVNCFYEGAKLDSRNIVIFGAR--EIEVEERKIIEKTGVKIVYYD : 193
H.sap ARG1  : VSDGYSCVTLGGDHSLAIGTISGHARHCPDLCVVWVDAHADINTPLTTSSGNLHGQPVSFLLRELQDKVPQLPGFSWIKPCISSASIVYIGLR--DVDPPEHFILKNYDIQYFSMR : 197
T.the AAUH  : ----KWVVALGGDHSVTHPLVQAHREALGDFSLLHVDAHADLYPEWQG-SVYSHASPFYRLLTEGFP-------------------LVQVGIR--AMDRDSLRLARKKGVALFPAH : 185
D.rad AUH   : R----VPVFLGGDHSVSYPLLRAFADVP-DLHVVQLDAHLDFTDTRND-TKWSNSSPFRRACEALPN----------------LVHITTVGLRGLRFDPEAVAAARARGHTIIPMD : 205
E.col AUH   : ----KRMLSFGGDHFVTLPLLRAHAKHFGKMALVHFDAHTDT---YANGCEFDHGTMFYTAPKEGLID---------------PNHSVQIGIR--------TEFDKDNGFTVLDAC : 202
                                               ▲

B.sub AUH   : ---------VLEPLKEVLPKLAG-RPVYVTIDIDVLDPAHAPGTGTVDAGGITSKELLASVHEIARSEVNVKGADLVEVAP-VYDHSEQTANTASKIIREMLLGFVK--------- : 290
C.dif AUH   : ---------GIDTFENIVNMLNG-KNIYLTIDLDVLDASVFPGTGTPEPGGVNYREFQEIFKIIKNSNINIVGCDIVELSP-DYDTTGVSTVIACKILRELCLIISDKIK------ : 285
T.kod AAUH  : DY-------SFDAFVDLVEALP--EPVYLSIDIDVFDLSMVPSTGTPEAGGLRFWEVVEAIEWLVEKKE-IAGFDIMEVAG-EKLG-DPTALTAAKLLFYSIGAMAKFGR------ : 288
T.the ARG   : EVDRLGVARIAEEVLKHLQGLP----LHVSLDADVLDPTLAPGVGTPVPGGLTYREAHLLMEILAESGR-VQSLDLVEVNP-ILDERNRTAEMLVGLALSLLGKRIF--------- : 290
B.ant ARG   : EIDRMGMTAVMEETIEYLSHTDG---VHLSLDLDGLDPHDAPGVGTPVIGGLSYRESHLAMEMLAEADI-VTSAEFVEVNM-ILDERNRTATTAVALMGSLFGEKLK--------- : 297
F.nuc ARG   : DILRKGIDNVLDEVKDYLKIDN----LHISIDMNVFDPEIAPGVSVPVRRGMSYDEMFKSLKFAFKNYS-VTSADITEFNP-LNDINGKTAELVNGIVQYMMNPDY---------- : 293
H.sap ARG1  : DIDRLGIQKVMERTFDLLIGKRQ-RPIHLSFDIDAFDPTLAPATGTPVVGGLTYREGMYIAEEIHNTGL-LSALDLVEVNP-QLATSEEEAKTTANLAVDVIASSFGQTREG---- : 306
T.the AAUH  : RIHR-----EGLPLDEILRALG--KRVYISLDFDALDPSLMPSVGTPLPGGLSYRQVVDLLEAVFREKE-VVGMDFVELSPNGQFHAEMTAAQLVYHAIGLKGLQAGWLSREVDHI : 293
D.rad AUH   : D--------VTADLAGVLAQLPRGQNVYFSVDVDGFDPAVIPGTSSPEPDGLTYAQGMKILAAAAANNT-VVGLDLVELAP-NLDPTGRSELLMARLVMETLCEVFDHVL------ : 305
E.col AUH   : QVND----RSVDDVIAQVKQIVGDMPVYLTFDIDCLDPAFAPGTGTPVIGGLTSDRAIKLVRGLKDLNI--VGMDVVEVAP-AYDQSEITALAAATLALEMLYIQAAKKGE----- : 306
                                    ▲
```

Supplemental Figure S4. Alignment of ancestral- and extended-form ADC, ODC, LDC and O/LDC AAT-fold decarboxylases. The alignment is based on sequences used to construct the ML tree in Figure 6. For ease of presentation, the alignment below was made with ClustalW. Sequence names ending with a or -a are ancestral forms, those ending with w or -w are the extended forms. Insertions in the extended forms relative to the ancestral forms are shown in the ancestral forms as red dashes. Sequence names include UNT, untested; ODCi, LDCi, ADCi, inducible forms; ODCc LDCc, constitutive forms. The corresponding species name, phylum, protein accession number (GenBank) and complete protein amino acid size for each sequence is shown in Figure 6.

```
                             *        20         *        40         *        60         *        80         *        100
StlugYaaOa : --PINYKLSQLINEKAISMHVPGHKNMTIG-----------TLHELDFKFDMT-EITGLDDLHHPEDIILNSQHNMSK-HEDYDAFILVNGTTSGILATIQGFS :  89
StaurYaaOa : --PILNKLESLNQEEAISLHVPGHKNMTIG-----------HLSQLSMTMDKT-EIPGLDDLHHPEEVILESMKQVEK-HSDYDAYFLVNGTTSGILSVIQSFS :  89
LimonYaaOa : --PLVERLDAHAKSCPISLHVPGHKSGAIYP----------DAWQKLLKWDVT-EITGLDDLHHPEDVILEAELLAECYGSKKSYFLVNGTTSGGNLAVIMATL :  91
BasubYaaOa : --PLYKALIQHARRNSHSFHVPGHHNGDVFFDD------AKSIFDPLLTIDVT-ELAGLDDLHHPSGVIKEAQELASQLYGSAESFFLVNGTTVGNLAMILSVC :  95
CldifYaaOa : -MFIINELNKIVNEEVISFHMPGHKKGQVYKKL-----GYKDILKNLYKMDTT-EILGTDNLHFPEGIIKNSQKKASRVFKSDYTCYLVNGSSCGIQSAIMSVC :  97
CrserUNT-a : -PFFDAVQ-SYIGKHRAGLHMPGHMSGQGIHPR-----LKEFWGENVFLSDLT-EVEGLDYLHRPIGVVKEAQELAAEAYGAIESFFLVNGSTVGNQAMILSTC :  96
CoproUNT-a : -PYYSGLL-AYIAKHRASFHMPGHKRGRGVHPD-----FKAFLGENVFLLDLT-EVQGVDYLHKPTGILREAMDLLADAYGAKESFFLVNGSTVGNQAALMAVG :  96
CaexiODC-a : -PLYEAVS-KYIKRKMIPFHMPGHSQGKGAPKI-----LKRLFGDKFFDFDLT-EVSGLDYLHYAQGVIREAENLASSLYGTEATIFLVNGTTAGVHAMILDSV :  96
UTG1RsUNTa : -PLFDTLL-AHAKRDVVSFHCPGHKNGKGIDRE-----LKDYTGEEVYKFDVT-VFDEVDSLHDPVGSIKKAQELMAQAYGVEHSFFLVNGSSVGNIAMFLAAC :  96
ElubaUNT-a : -PLFDVLY-NRSLRKVVSFHTPGHKNGESIEKR-----LKSFTGRNVYYFDVT-VFPEVDSLHDPTGPIKKAQELAASAYGVDHSLFMVNGSSGGNIAMFLATM :  96
MeoxyUNT-a : -PLFDAMV-NLAESRKVSFHTPGHKSGKGISTR-----FRKFVGPKIFTIDLT-TLDEVDCLQRPVGVIKEAQELAAEAYGADHSFFLLNGTTGGNHAMIVSTA :  96
LeferLDC-a : -PLFDAMV-ALAESRKVSFHTPGHKSGKGISTR-----FRKFVGPKVFSIDLT-CLDEVDSLQKPRGVIREAQELAADIYGADQSFFLVNGTSGGNQAMILSVM :  96
BacihADC-a : -PLFTSLV-NHAKKKPIQFHIPGHKKGQGMDPE-----FREFIGDNALSIDLI-NIAPLDDLHSPKGVIKQAQELAAEAFGADHTFFSVQGTSGAIMTMVMTVC :  96
PesimADC-a : -PLYTALL-QHAKRNPVQFHIPGHKKGKGMDAD-----FRNFIGENALSIDLI-NIAPLDDLHQPKGIIKQAQDLAAEAFGADHTFFSVQGTSGAIMTMVMTVC :  96
BasubADC-a : -PLYTGLK-KHASRQPVQFHIPGHKKGAGMDPE-----FRQFIGENALSIDLI-NIEPLDDLHAPKGIIKQAQDLAAEAFGADHTFFSVQGTSGAIMTMVMAVC :  96
FunucODC-a : -PLFTVLKDEYVRRNILPFHVPGHKRGKGVDEE-----FYNFMGEAPFSIDVT-IFKMVDGLHHPKSCIKESQELVADAYGVKHSFFAVNGTSGAIQAMIMSVV :  97
FuulcUNT-a : -PLFSVLKDEYVGRDILPFHVPGHKRGKGVDKE-----FYDFMGNGPFSIDVT-IFKMVDGLHQPKSCIKEAQELAADAYGVKKSFFAVNGTSGAIQAMIMSVM :  97
IlpolUNT-a : -PLFSVLKDVYAKREIVPFHVPGHKRGAGMDKE-----FFDFIGKNAFSIDVT-IFKMVDGLHQPKSCIKEAQDLAADAYGVKKSFFAVNGTSGAIQAMIMAVV :  97
FunecODC-a : -PLFSVLKEEYAGNNTLAFHVPGHKRGKGADEE-----FINFVGEGPFTIDVT-IFPMVDGLHHPHGCIKEAQELAADAYDVKHSFFAVNGTSGAIQAMIMSVV :  97
ClbotODC-a : -PVFDALM-EYVNRKTIPFHVPGHKKGVGMDEA-----FKNFIGENPFKIDVT-VFKLVDSLHHPTGAIKKAQELAADAYGSDAAFFSIHGTSGIQTMVMSAC :  96
CltetUNT-a : -PLFDALM-EYVNRQTIPFHVPGHKKGVGIDEE-----FKNFIGENPFKIDVT-VFKLVDSLHHPTGPIKKAQALASDAYGSDASFFCIHGTSGAIQAMIMSVV :  96
ThethUNT-a : -PLFDALM-EYVNNNTIPFHVPGHKKGVGMAKK-----FLDFVGKNVLSMDVT-VFQQVDSLHKPTGPIKYAQELAAEAFGADATFFSIHGTSGAIQAMILSVM :  96
ThtheOLDCa : -PLFDALM-EYVNNNTIPFHVPGHKKGNGMAKK-----FFDFVGKNVLSMDVT-VFEQVDSLHKPTGPIKKAQELAADAFGADATFFCIHGTSGAIQAMILSVM :  96
LebucUNT-a : -VLFDALK-NHLSNRVVRFDVPGHKGGRG-NKE-----FRDFIGLDAMQMDVN-SMKPLDNLCHPTSVIKEAQDIAAEAFGAKEAYFMVSGTTGAVQAMIMATC :  95
BrmurUNT-a : -PIVEALK-QYRKKRIVSFDVPGHKQGKS-TKV-----LIDVLGEKCVQIDYN-SSKPLDNATHPIGVIKEAQELMAEAFKAKESFFMVGGTTSAVQAMIFTAC :  95
StpneADC-a : -PIYESLV-KLRKKRIVPFDVPGHKRGRG-NPE-----LVELLGEKCVGIDVN-SMKPLDNLGHPISIIRDAEELAADAFGASHAFLMIGGTTSSVQTMILATC :  95
SerumADC-a : -PVYEAML-ELRKRRVVPFDVPGHKRGRG-NPE-----LVDFLGEKCVGVDVN-SMKMLDNLSHPISVIKEAEELTADAFGAQHAFFMVHGTTSAVQAMVLSTV :  95
HutheADC-a : -PVFDAVK-RYVESNVIQFHVPGHKQGAG-LEE-----LREYIGETALKMDAN-GMEDLDFANNPTGVIYESEMLAAQAFGAQHAYFLVNGTTSGVQAMIMSAC :  95
PsychADC-a : -PLFDALK-NYHKTDIVSFDVPGHKKRF--QNE-----FSDYVGHKIIELDTN-SMKELDILNNPTGVIREAHQLMADAYSADEAFFLVNGTSGIQTMVMSAC :  94
CldifADC-a : -PLLESLK-EYSEKDIACFDVPGHVKNRG-VAI-----LNKYLGESLMKMDIN-SSPTVDNVSAPNGIIKNAQDLLAQAYMADEAFFITNGTTQAIHAMILSVI :  95
AclaiUNT-a : -PFYDKIK-SYGQSHTVALDVPDHKLGAI-QND-----FRNYVGEHLFLLDAN-APRGLDNLSKPKGVIKEAQALAADAFGADHAYFLVNGTSQGIMAMIMTAC :  95
MeblaADC-a : -PIIDALR-EFDQKNITSYDVPGHKRGIR-EDD-----FQKLVGKEVYLMDVN-SPVGMDTFAHPKGVIKHSLELAAEAYKADNAFYLVNGSSSGVQIMIMTVC :  95
ChvarUNT-a : -PLLDAVVERGERFQEVPFHVPGHKRGGTPPC-----LGRMLAG-AERYDLT-ELEGLDILSSPSGPILAAQQLAAAWGADATWFLVNGSTAGIHAAVMATC :  96
PhpatUNT-a : APLLSALRECAE-MDAASFHFPGHRRGSGAPPV-----MEALIGKSAFAADLP-ELPELDNLHAAEGVILEAQCKAARLFGAEQTWFLVNGSTCGIQAAVMATC :  97
NopunUNT-a : TPLLDALKANAA-RPHAPFYTPGHKQGEGIPQP-----LADLLGKAVFRADLT-ELADLDNLFAPQGVIQEAQQLAAEAFGASQTWFLVNGSTCGIEAAILATC :  97
SyspeUNT-a : ---MMALLPQLTRRLGQPLFLPAHGRGAALPDG-----LRQLLRRRAGVWDLP-ELPALGGPLEADGAIADSQRSAAAQMGVARCWYGVNGATGLLQAALLAMV :  95
PachrUNT-a : ---MKFYEFNIKRKSKIALHLPAHGRGPGLAPG-----LKWLLNCRPATWDLP-ELPAIGGPLESKGVVAYSQRIMAERWGADHCWYGVNGATSMLQVALMAMT :  95
StcoeUNT-a : --PVLEALVEYRRAGRLSFTPPGHKQARGADPA-----VREVLGDAVFHGDVL-ATAGLDDRLTRGRVLQRAQELMADAVHADHTFFTTCGSSLSVKAAMLAVA :  96
MysmeUNT-a : --PLLDALNEYHASNRYGYSPPGHRQGRGVDDR-----VLRVLGREPFLSDVL-ANGGLDDRRSSNGYLQKAEDLMAEAVGADTAWFSTCGSSLSVKAAMMAVA :  96
BoperODCcw : -PPFFRELVKYAQDGSYSWHCPGHSGGVAFLKSPVGQMFHQFFGENMLRADVCNAVDELGQLLDHTGPVAESELNAARIFHADHCFFVTNGTSTSNKVVWHANV : 103
RalstODCcw : -PPFFKALIDYAQDSSYSWHCPGHSGGVAFLKSPVGQVFHQFFGENMLRADVCNAVDELGQLLDHTGPVAASERNAARIFGSDHMFFVTNGTSTSNKMVWHANV : 103
LebifUNT-w : -PPFFRALTQYAHDGSYSWHCPGHSGGVAFLKSPVGQMFHQFFGENMLRADVCNAVDELGQLLDHTGPISASERNAARIFQCDSLYFVTNGTSTSNKIVWHSTV : 103
PseaeLDCcw : -PPFFRALVEHTAQSNYSWHTPGHGGGVAYRKSPVGQAFHQFFGENTLRSDLSVSVPELGSLLDHTGPLAEAEDRAARNFGADHTFFVINGTSTANKIVWHSMV : 103
EscoADCi-w : -PPLFSALMKYSDIHEYSWAAPGHQGGVGFTKTPAGRFYHDYYGENLFRTDMGIERTSLGSLLDHTGAFGESEKYAARVFGADRSWSVVVGTSGSNRTIMQACM : 103
FuvaUNTi-w : -PPLAKAVFNYNKVAEYSWAAPGHQGGVGFLKTAVGKKFFDYYGENLFRTDTGIERTSLGSLLDHTGAFLDSEKNIARIFGSDRSYSVLVGTSGSNRTVFQACL : 103
MebooUNTiw : -PPFFGELVKFSKDFEYSWHTPGHAGGTAFRKTPAGRRFFEFFGEQLFRSDLSISVGELGSLLDHSGPVGEAERYAAKVFGADMSYFVTNGTSTANKIVFFGRV : 103
StambUNT-w : -PPFFKALRRFDDAHEYSWHTPAHSGGVAFLKSPVGRAFHDYFGERLLRSDLSISVEELGSLFEHTGPIGEAEHNAARVFGSDRTYFVLHGDSTCNRLVGHFSV : 103
AcspeUNT-w : -PPYFRELKKYTDQGAYSWDAPGHMGGVAYLKHPIGMEFHKFFGENIMRSDLGISTSPLGSWLDHIGPPGESERNAARIFGADWTFFVLGGSSTSNQIVGHGVI : 103
VivadUNTiw : -PPYFKTLKDFTQDGDYYWDCPGHMGGMAYLKHPIGVEFFNFFGENMFRADIGVATSEMGDYLIHAGPVKKSEEIAAKTFGADWTFYTVAGSSGSNRIVAQGAV : 103
EscoLDCi-w : -PPLTKALFKYVREGKYTFCTPGHMGGTAFQKSPVGSLFYDFFGPNTMKSDISISVSELGSLLDHSGPHKEAEQYIARVFNADRSYMVTNGTSTANKIVGMYSA : 103
EscoLDCc-w : -PPFTKALFTYVKERKYTFCTPGHMGGTAYQKSPVGCLFYDFFGGNTLKADVSISVTELGSLLDHTGPHLEAEEYIARTFGAEQSYIVTNGTSTSNKIVGMYAA : 103
PypisUNT-w : -PPFTRALFKFAAAKKNTFCTTGHLLGSAFRHHAMGWAYYNFYGPNAFRADTSVSVPDMGSLLEHTGAHKDAEELIARAFNADRSYIVTNGTSTANKIVGMYCV : 103
FratuLDCcw : -PPLTYELFKYSKSFNSAFCTPGHQGGYGFQRSAVGALFYDFYGENIFKTDLSISMKELGSLLDHSEAHKDAEEYISKVFKSDRSLIVTNGTSTANKIVGMYSV : 103
ElminUNT-w : -APFFKALKNFALTKADMWSTPGHGGGQGLKSSVLGREFYEFFGKNIFRADISSSVPSLGTILDHEGAAEDAEKNAAEIFGADTTFFVINGTSTANKVVFFATC : 103
StlugODCiw : -PPFFNTLADYVERGNVQFDCPGHQGGQYFAKHPAGRQFYKFFGENIFRADICNADVDLGDLLIHEGPAVDAQKHAAKVYNADKTYFVVNGSSTSNNITITSAI : 103
StepiODCiw : -PPFFNTLANYSDRGNIQFDCPGHQGGQYFAKHPAGKQFYKFFGENLFRSDICNADVDLGDLLIHEGPAVEAQKHAAKVYNADKTYFVVNGTSTSNNIAITSAI : 103
OeoenODCiw : -PPFFKALKAYVERGNIQFDCPGHQGGQYFRKSPAGREFYNFYGENIFRSDICNADVDLGDLLIHEGPAMDAEKHAARVFNADKTYFVVNGTTTSNNIAITAAV : 103
EscoODCi-w : -PPFFRALVDYVNQGNSAFDCPGHQGGEFFRRHPAGNQFVEYFGEALFRADLCNADVAMGDLLIHEGAPCIAQQHAAKVFNADKTYFVLNGTSSSNKVVLNALL : 103
EscoODCc-w : -PPFYDTLTQYVEMGNSTFACPGHQHGAFFKKHPAGRHFYDFFGENVFRADMCNADVKLGDLLIHEGSAKDAQKFAAKVFHADKTYFVLNGTSAANKVVTNALL : 103
DiinvUNTiw : -PPFFKQLMEYVERGNTQFDCPGHQGGAYFRKHPAGRIFYNFYGENTFRADLCNADVAMGDLLIHEGPALEAQRHAARVFHADKTYFVLTGTSGANKVVLDALL : 103
VedisUNT-w : -PPFFRALSDYVGDENSQFDCPGHQGGQFFYKHPTGSAFYNFFGENIFRADLCNADVKLGDLLIHEGYAYDAQAHAAKVYNADKTYFVLNGTSSANKVVLNALL : 103
LabacODCiw : -PGFLRDLINFADEKPVSYTTPGHHNGQFYEKHPAGVVFKKFFGDNMLRADVSDTVAELGDTLTHGGTPLDSEQEAAQAFNADKVYYVTNGTTSSNTICANAVL : 103
GrbetUNT-w : -TPFFGQLVDYAEQGNMLWTCPGHNGGVFYRRSPIGRIFVEHLGEAIFRDDLDNSVLQLGDLLTHEGPALKAQREAATIFGAERTYFVLNGTSTSNKIALNALL : 103
TethUNT-a  : -KQIFEAIEEFIQSDPTQLQISYHSGRTNSQPRLN--KLIQTYGFKKVDATWE-CMQNIAGLQFSGSRIGDLGEVFHQTFNSKLSLFVQNGSSMGNQVIALALA : 100
               p                      pgH  g               g       d                g           a                 G 3
```

```
                  *       120         *       140         *       160         *       180         *       200
StlugYaaOa : AVDGRYLIARNVHKSVFH-GLDLTHQSADILPMQESLQTHHYYGPQTRDFDSEQY---------------------KLAVVTYPNYYGECFDINSCISKLHAAN : 171
StaurYaaOa : QKKGDILMARNVHKSVLH-ALDISQQEGHFIETHQSPLTNHYNKVNLSRLNNDGH---------------------KLAVLTYPNYYGETFNVEEVIKSLHQLN : 171
LimonYaaOa : KRGEKVLVPRDAHKSILH-GIELAGGEPIFLTPATNKEVGVASGVTTELLEETLHNHP----------------DVKLCIFTYPSYYGTTFNLQKCIRIAHDFG : 178
BasubYaaOa : EPGDTILVQRNCHKSVFH-AVDLSGAEPVYLAPDVDSAMHVPTHVPLGTIKEALEAYP----------------DAKGLVLTNPTYYGHSADLTEIITEAHHYG : 182
CldifYaaOa : SPKSKIITNRDCHQSVIN-GCILGDIDIEYVPCEISEETNVLKGISISNVIDIIDKNL----------------DAKAVLLTYPTYYGMTYDLEYICNYAHSKK : 184
CrserUNT-a : DPNDRVIVQRNSHRSMYG-ALILNELRPIYLEAEEDRLLQFQTFPSKKTFARVLRDHP----------------EAKTVFVTNPNYFGISSDIDYYVQKAHATG : 183
CoproUNT-a : GPGKKILIARNSHRSVVG-GLILNGSIPVYLPVNVDPELGITTSVSPEVLREYLDKH-----------------DVDAVFLTNPNYYGVSGNIRELVDIVHEHG : 182
CaexiODC-a : RENEKIIIGRNSHRSVIG-GILLAKAKPVFVQPEFNEEFGIITNVTPESIKKAITENP----------------DAKAVLVTTPNYYGLQGRIKDIIDIVHENG : 183
UTG1RsUNTa : DSGDSVIVSRSSHKSIMA-GIIMSGVWPIWIQPKIDQNLDLIFNSTYNQVKDALDRYP----------------EAKAVFITSPTYNGIVTELSRIVDLCHRRG : 183
ElubaUNT-a : RAGESVIVSRNAHKSVLA-GVILSGVWPIWIQPEIDEQNKIINNSSARQIEDALDKFP----------------EARAVFITSPTYNGVVADLFKIAEICHRRG : 183
MeoxyUNT-a : SPGDEILIARNAHKSILT-GIILSGATPRFFLPAFDRELGMLLNVTVEEVGRTMAQYP----------------KAKILSLTSPNYYGVTADLRAIVSMAKRRG : 183
LeferLDC-a : KPGEPILLTRILHKSVVS-GLIMTDCKPIFIPLQSTPDCDLLLNDRTDTIIQAMNRHP----------------EAKKLFLTSPNYYGVTLDLPTIIQEAHSRG : 183
BacihADC-a : GPGDKILVPRNVHKSIMS-AIVFSGAIPIFITPEIDKNLGISHGITIDSVEKALRENP----------------DAKGLLVINPTYFGVSADLKKIVELAHSYQ : 183
PesimADC-a : GPGDKIIVPRNVHKSVMS-AIVFSGAVPIFINPEIDKDLGISHGITVDAVEKTLREHS----------------DAKGLLVINPTYFGISADLQKIVEVAHSHQ : 183
BasubADC-a : GPGDKIIIPRNVHKSIMT-AIVFSGAVPIFIHPEIDNELGISHGITLESAKRALTEHP----------------DAKGLLVINPTYFGVAADLKSIVELAHSFD : 183
FunucODC-a : KAGEKILVPRNVHKSVSA-GIILSGSEPVYMNPEIDENLGIALGVKPQTVENMLKQDP----------------DIAAVLIINPTYYGVATDIKKIADIVHSYD : 184
FuulcUNT-a : KAGDKILVPRNVHKSVSA-GIILSGSEPIYMNPEVDEELGIAHGVRPQTVENMLKQHP----------------DIKAVLIINPTYYGVATDIKKIADIVHSYD : 184
IlpolUNT-a : KSGEKILVPRNVHKSVTA-AIILSGAEPIYMNPEIDDDLGIAHGVKPETVEKMLKQHS----------------DIKAVLIINPTYYGVATDIKRIADIAHSYD : 184
FunecODC-a : NPGEKLLVPRNVHKSVSA-GIILSGAKPVYMNPEIDEELGIAHGVRPQTVAEMLAQDS----------------EIKAVLIINPTYYGVATDIKKIAEIVHQYD : 184
ClbotODC-a : GDGDKIIIPRNVHKSVTA-GIILSGSVPVYMQPELDKKVGIAHNVTPETVEKTLKENP----------------DAKAVLIINPTYYGVSTDIKKIAEIVHSYN : 183
CltetUNT-a : KSGDKIIIPRNVHKSVTS-GIILSGATPIYMQPELDKRVGIAHGVTPETVEKTLKENP----------------DAKAVLIINPTYYGVATDIKKIADIVHNYD : 183
ThethUNT-a : GEEEKIIVPRNIHKSVTS-GIILSGAIPVYMQPEIDKNIGVALNVTPETVERTLKEHP----------------DAKAVLIINPTYYGVSTDIVKIAEIVHDYG : 183
ThtheOLDCa : GENEKIIVPRNIHKSVTS-GIILSGAIPVYMQPEVDKNVGVALNVTPETVEEALRNNP----------------DAKAVLIINPTYYGVSADIVKIAEIVHDYG : 183
LebucUNT-a : KAGDKIIIPRNVHRSAIN-AMVICGAIPVYINPGLNKKLGISLGMSINDVKKAIHENP----------------DAKAILVNNPTYYGICSDLKSIVKLAHENN : 182
BrmurUNT-a : KYGDKIILPRNVHKSAIN-ALVLCGAVPIYVNPGVNKELGISLGMSVENIKKAIEKNP----------------DAKAIFVNNPTYYGICSDLKKIVELAHKNN : 182
StpneADC-a : KAGDKIILPRNVHKSAIN-ALVLCGAIPIYIEMSVDPKIGIALGLENDRVAQAIKDHP----------------DAKAILINNPTYYGICSDLKGLTEMAHEAG : 182
SerumADC-a : RAGEEILLPRNVHKSVIN-ALVLCGGIPVYVPVRVNHQIGIATGMALSDVERAIRQHP----------------QARPSLSIIRLTMALPLDLKGIVELAHQAG : 182
HutheADC-a : EPGDKIILPRNAHKSTIG-GIILSGAVPVYVQPEINEKLGIAMGITVESLKKAIKENP----------------HAKAVFIINPTYYGIASDLKSLVRIAHRYE : 182
PsychADC-a : NPGDKIILPRNVHKSAIN-ALILSGATPVYIDPEVDETLGISLGISVEKIKEALIKDP----------------DIKAVFIINPTYYGAVSDLEEIIKICHEKN : 181
CldifADC-a : KPGEKVLLPRNIHKSVIN-ALILCGGIPIFIQPEFDEKLGISLNITLEKVRTEIEKDC----------------NIKALFFLNPTYYGVCADLESIIELCHKNN : 182
AclaiUNT-a : RAKEKIIIPRNVHKSAIN-GLILSGAMPIFMKPYIDSDLGIANGIDIEELKETIAEHP----------------DAKAVFVINPTYFGVTSNLEEVVKIAHAHD : 182
MeblaADC-a : YDGEKILIPRNAHKSVTN-SLILSGAVPVFMEPEINQNFAIASGMTLKTIQKYVEANP----------------DAKALFLVYPTYYGACADLEQIIEYAHSKD : 182
ChvarUNT-a : GPGDALLARNAHLSAFN-AMVLAGCTPVYAQPCCDAHLGIAHHVAPAALEAAFEAAARQEL-------------RVGAALVVSPTYFGVLSDIAGLAAVCHARG : 187
PhpatUNT-a : APGEYLILPRNVHMSAVS-AMVLSGALPKYVSPISDSTWGVAHGVQIAQFEAAIREIRSTGG------------KIGALLVVSPTYFGICSLIRDLAQVCHAQD : 188
NopunUNT-a : GTGDKIILPRNVHSSAIA-GLILSGAIPIFLNPEYDPVLDIAHSITPNALESALQQHP----------------DAKAVLTVYPTYYGVCGDLSAIANITHQYN : 184
SyspeUNT-a : RPGERVLLPRNVHRSLIQ-ACVLGDLRPVLFDLPFQSDRGQPAPADSAWIKRVLEALPRDGA------------PIRAAVLVHPTYQGYANDPTAVIQRLQQQG : 186
PachrUNT-a : SPGDRVLMPRNIHRSLIN-GCILARLKPILFNIPFDPYTGLWQLPDAENFEKILIKIG----------------HIAAVVLIHPTYQGMAGNLTSLVEKSHQKG : 182
StcoeUNT-a : GPHEKLLIGRDAHKSVVS-GLILSGIEPVWVEPRWDAERHLAHPPSAESFEEAFGAHP----------------DAKGALITSPTPYGASADLRAVAEVCHGRS : 183
MysmeUNT-a : GGDGSLLLSRDSHKSVVA-GLIFSGVMPRWITPRWDADRHLSHPPSPEEVEETWRRYP----------------DAAGALIVSPSPYGTCADLAGIADVCHARG : 183
BoperODCcw : AAGDVVVVDRNCHKSILH-AITMTGAIPVFLRPTRNHLGIIGPIPLDEFDPENIRRKIEANP----FAREALNKKPRILTLTQSTYDGVIYNVEMIKEKLGTYV : 202
RalstODCcw : APGDIVVVDRNCHKSILH-AIMMTGAIPVFLMPTRNHYGIIGPIPQSEFEPETIARKIAEHP----FASQAANKKPRILTLTQGTYDGVLYNAERIKHMLSTEI : 202
LebifUNT-w : APGDVVIVDRNCHKSILH-AITMTGAIPVFLMPTRNHFGIIGPIPKSEFTWENIKKKIAEHP----FAKEVKGN-PRILTITQSTYDGILYNVEDIKSELDGKI : 201
PseaeLDCcw : GREDLVLVDRNCHKSILH-SIIMTGAIPLYLTPERNELGIIGPIPLSEFSKQSIAAKIAASP----LARGREPK-VKLAVVTNSTYDGLCYNAELIKQTLGDSV : 201
EscoADCi-w : TDNDVVVVDRNCHKSIEQ-GLMLTGAKPVYMVPSRNRYGIIGPIYPQEMQPETLQKKISESP----LTKDKAGQKPSYCVVTNCTYDGVCYNAKEAQDLLEKTS : 202
FuvaUNTi-w : TDKDTALIDRNCHKSIEQ-GLILTGAKPVYMTPTRNRYGIIGPILPDQMK------KVTEV-------------APKYVVVTNCTYDGVCYNAAKAEDIFQGNA : 187
MebooUNTiw : TENDVVLVDRNCHKSAEH-ALTMTHSVAVYMVPTRNRYGIIGPIPPSDMTPAAIRKKIAACP----LAKNLPDKTPVHAIVTNSTYDGLCYHAVEVEKELGKSV : 202
StambUNT-w : TRDEIALVDRNCHKSILH-GLVVSGARPVYLVPTRNGYGLAGPLPPTELAADSVAERIARSP----LTADAVSPRAQYAVLTNSTYDGVCYASPTVARAFASST : 202
AcspeUNT-w : AQDDIVLADANCHKSICH-SLTITGARPVYFKPTRNGYGMIGLVPIKRFSPENVQALIDKSP----FCAGAPVKKATYAVVTNSTYDGLCYDVNRVVEELAKSV : 202
VivadUNTiw : GANEITVVDRNCHKSLNH-GLTLCGARPVYMKPTRNGYGLIGPIPARRMSKEHIKKLIKASP----IARDAVSQEPTYAVVTNCTYDGFCYNAGKVVDALKDSV : 202
EscoLDCi-w : PAGSTILIDRNCHKSLTH-LMMMSDVTPIYFRPTRNAYGILGGIPQSEFQHATIAKRVKETP----NAT-----WPVHAVITNSTYDGLLYNTDFIKKTLD--V : 195
EscoLDCc-w : PSGSTLLIDRNCHKSLAH-LLMMNDVVPVWLKPTRNALGIIGGIPRREFTRDSIEEKVAATT----QAQ-----WPVHAVITNSTYDGLLYNTDWIKQTLD--V : 195
PypisUNT-w : SQGDTVLIDRNCHKSMTH-LLMMCDVVPIYLLPTRNAYGMIGGIPADEFTSEAIHYKLSQRD----DAT-----WPTYAVISDSTYDGLLYDCSWIKANLP--V : 195
FratuLDCcw : ADGDTILVDRNCHKSVTH-LMMMVDVNPIYLKPTRNAYGIIGGIPKKEFKRETIQEKIDNSN----IADK----WPEYAVVTNSTYDGILYNTDTIHRELD--V : 196
ElminUNT-w : APGDIVLVGRNCHKSIMH-SIILNEVTPIYLKPAKNSYGLIGPVPQSEFTHEAIKQKIKESP----IVTNKRIRKVQLTVLTNSTYDGLIYDVDKIKEKMSALT : 202
StlugODCiw : MPDDLVLFDRNNHKSVYISALVKDGGRPVYMQTARDSYGFIGGIHEHDFDEEYLRQEVAKV----DPERAKWDRPFRLAVIQLGTYDGTIYNARQVVDKIGHLC : 203
StepiODCiw : MPDDLVLFDRNNHKSAYNSALVKDGGRPVYMQTSRDSYGFIGGIYQEEFDEERLRERAAKV----DPERAKWERPFRLAIIQLGTYDGTIYNAKQVIEKIGHLC : 203
OeoenODCiw : APGDLVLFDRNNHKSVYNAALVQAGGRPVYLETSRDSYGFIGGIYSKDFDEKSIREKIAKV----DPEKAKAKRPFRLAVIQLGTYDGTIYNAKQVVERIGHLC : 203
EscoODCi-w : TPGDLVLFDRNNHKSNHHGALLQAGATPVYLETARNPYGFIGGIDAHCFEESYLRELIAEV----APQRAKEARPFRLAVIQLGTYDGTIYNARQVVDKIGHLC : 203
EscoODCc-w : TRGDLVLFDRNNHKSNHHGALIQAGATPVYLEASRNPFGFIGGIDAHCFNEEYLRQQIRDV----APEKADLPRPYRLAIIQLGTYDGTVYNARQVIDTVGHLC : 203
DiinvUNTiw : APGDIVLYERNNHKSISYGALIQAGAVPVYLETARNPFGSIGGILEHCFNEEYIRMVIAEK----DKKKAKAKRPVRVAVIQLGNYDGCIYNARQIVEKIGHLC : 203
VedisUNT-w : TPGDIILYDRNNHKSICHGGLVMSGATPIYLETARNPFGSIGGILDHCFDESYIRELVAEK----SPEKANAKRPIRLAVIQLGTYDGTIYNARQVVDKIGHLC : 203
LabacODCiw : SEGDLVLFDRNNHKSLYNSALIMTGAKPVYLPTSRNPLGLIGPVISDDLDEEKIRAEIAKV----DPERAKAKRPFRMAVIQLETFDGVFYNAKWLLDKIGKLC : 203
GrbetUNT-w : AEGDLVLFDRNNHKAAHHGALLLSGAVPVYIDTARNPHGMIGPMRRDALNEEALREAIRTHPLVRDPEAWRKPRPFRVAMIEQCTYDGTIHSAEEVLATIGHLC : 207
TethUNT-a  : H--KKVLIQANSHVSVYT-GLSLSNAEIVLLEPEFNQEYGIHMAVTADQIQQAIQQHP----------------DIYAVLLTSPSYEGLFADYQKIRDVIG--S : 183
                   6  r  H s            p                                                           g

              *       220         *       240         *       260         *       280         *       300         *
StlugYaaOa : IPVLVDEAHGAHFDL-------DG-FPTSALNMK----ADYVVQSYHKTLPALTMGSILFIHKQAPQ-------RDKVIQYLSYFQTSSPSYLVMASLEQANQF : 256
StaurYaaOa : IPVLIDEAHGAHFGL-------QG-FPDSTLNYQ----ADYVVQSFHKTLPALTMGSVLYIHKNAPY-------RETIIEYLSYFQTSSPSYLIMASLESAAEF : 256
LimonYaaOa : AVVFVDEAHGAHFLT-------SSEFPKSAVELG----ADVVVQSAHKTLPALTMGSYLHVVNDLPI-------FEKLAYYLQVFQTSSPSYLIMASLDAARKY : 264
BasubYaaOa : IPVLVDEAHGAHFIL-------GEPFPVSALKMG----ADIVVQSAHKTLPAMTMGSYLHLNSSCRIN------RDRVAEYLNRLQSSSPSYPIMASLDIARAY : 269
CldifYaaOa : MVVIVDEAHGAHLGL-------SERLPKTALEQG----ADIVVQSTHKTLPAFTQSSMIHIQGDRVD-------IDKILNMLRITESSSPSYLLLSSLELAVDI : 270
CrserUNT-a : RRILVDEAHGSHFHF-------HPDLPKSAVDAG----ADMVVQSTHKTLSGLTQTSMLHLGSRRVD-------KELVRATLTMLESSSPSYILMTALDVARMQ : 269
CoproUNT-a : LPIVVDEAHGAHFHF-------HQALPLSAVDAG----ADLVVQSTHKTLSSMTQSSWMHMNGDLVD-------VELLKAALRTLQSSSPNYVLMASLDVARMQ : 268
CaexiODC-a : MRALVDEAHGAHFPF-------NSKFPKSAIYLG----ADLVTQSAHKTLPTLTQTSFLHIPSKDVN-------VDRIEQILSIIESSSPSYIFMTALDVARRE : 269
UTG1RsUNTa : KTVLVDEAHGVHLLF-------NNQLPESAVSAG----ADLCVQSTHKILSAMSQGSVLHFNSKLVD-------FNRVKRVVSMLQTTSPNYLILATLDLARRQ : 269
ElubaUNT-a : KLLLVDEAHGAHLKF-------HKDLPVSAVETG----ADMCVQSTHKMLSAMSQGSMLHVNSDMVD-------INAVRQVVSLNQTTSPNYLTLASLDMARWQ : 269
MeoxyUNT-a : ILVLVDEAHGPHLHF-------HQALPTSALDAG----ADLCVQSTHKILGGMTQSSMLHLKGSDID-------HSRVVKLLLLLQSTSPSYILMASLDLARMQ : 269
LeferLDC-a : VTVLVDEAHGPHLHF-------HDDLPPSAMASN----ADLCVQSTHKIIGGMTQASILHAKKANID-------IERLTSVLRYLQTTSPSYILMASLDLARMQ : 269
BacihADC-a : IPVLVDEAHGVHIHF-------HDELPLSAMQAG----ADMAATSVHKLGGSMTQSSILNVKEGLVS-------ANRVQSIISMLTTTSTSYILLASLDTARKR : 269
PesimADC-a : IPVLVDEAHGVHIHF-------HEDLPMSAMQAG----ADMAATSVHKLGGSLTGSSILNVKTGLVS-------ANRVQAILSMLTTTSTSYILLASLDTARRQ : 269
BasubADC-a : VPVLVDEAHGVHIHF-------HDELPLSAMQAG----ADIAATSVHKLGGSLTQSSILNMREGLVS-------KDRVQSILSMLTTTSTSYLLLASLDVARKR : 269
FunucODC-a : IPLIVDEAHGPHLHF-------HDELPVSAVDAG----ADICTQSTHKILGAMTQMSLIHVNSDRVN-------VEKVKQILSLLHTTSPSYPLMASLDCARRQ : 270
FuulcUNT-a : IPLIVDEAHGPHLHF-------HDELPLSAVDAG----ADICTQSTHKIIGAMTQMSLLHVNSDRID-------TNRVQQILSLLHTTSPSYPLMASLDCARRQ : 270
IlpolUNT-a : IPLVVDEAHGPHLHF-------HEDLPISAVDAG----ADICAQSTHKIIGALTQMSLLHVNSDRVD-------QNRVQQILSLLHTTSPSYILMASLDCARRQ : 270
FunecODC-a : IPLIVDEAHGPHLHF-------HEELPTSAVDVG----ADICSQSTHKILGALTQMSLLHVNSERVK-------VERVREILSMLHTTSPSYPLMASLDCARRQ : 270
```

```
ClbotODC-a : IPLIVDEAHGPHLGF-------NDRLPVSAMEAG----ADMCAQSTHKIIGALTQCSLLQVKSKRID-------VNRVHQVLSLLQTTSPSYILMASLDCSRRQ : 269
CltetUNT-a : IPLIVDEAHGPHLAF-------NDTLPISAIEAG----ADICCQSTHKIIGALTQCSLLHVRSKSID-------VNRVQQMLSLLQTTSPSYILMASLDCARRQ : 269
ThethUNT-a : AILMVDEAHGPHLKF-------NEKLPISAMEAG----ADICAQSTHKIIGSMTQSSMLHVKSKRID-------INRVKQVMSLLQTTSPSYILLASLDVARMQ : 269
ThtheOLDCa : AILMVDEAHGPHLKF-------NEKLPISAMEAG----ADICAQSTHKIIGSMTQSSMLHVKSKRID-------INRVKQVMSLLQTTSPSYILLASLDVARMQ : 269
LebucUNT-a : MLVLVDEAHGAHFSF-------DENLPISGMAAG----ADMAAISMHKTGGSLTQSSILLS-GERIN-------ADYVRQIINLTQTTSSSYLMTSLDVARKN : 267
BrmurUNT-a : MLVLADEAHGTHFYF-------GDNLPISAMEAG----ADMSCVSLHKTGGSLTQSSALLI-NNNVN-------GNHVRQIINLTMTTSPSYILMSSLDIARSE : 267
StpneADC-a : MMVLVDEAHGAHLHF-------TGKLPISAMDAG----ADMAAVSMHKSGGSLTQSSLLLI-GEQMN-------PEYVRQIINLTQTSTSASYLLMASLDISRRN : 267
SerumADC-a : MKVLVDEAHGTHLYF-------GDNLPISGMEAG----ADLAAVSMHKSGGSLTQSSILC-GKDVD-------AEYVQQIINLTQTTSASYLLISSLDLSRRN : 267
HutheADC-a : MAVLVDEAHGAHMSF-------HDDFPLTAMEVG----ADMSAVSTHKTGGSLTQSSLLLRGNMIS-------PERVKQVLNLTYTSSASYLLMCSLDIARKQ : 268
PsychADC-a : MVVLVDEAHGAHFPF-------HEKFPKNAMALG----ADMSAVSIHKTGGSLTQSSVLFLNHRRIK-------QERVKNMLNLTQTTSASYLLMTSLDVARKT : 267
CldifADC-a : VLVLVDEAHGAHFPF-------HLDLPPSAISLG----ADMVAVSIHKTGGALTQSSALLLNRDNVS-------FEKVLQSINVLQSTSASYLLMASIDGARVN : 268
AclaiUNT-a : MLVLCDEAHGAHFNF-------NDKLPISAMAAG----ADMSAGSIHKTVGSLTQSSILLIKGDRIE-------PSRVQSTLNILQSTSPSSLFMASLDASRSH : 268
MeblaADC-a : IVVMVDQAHGAHFSF-------SSALPKSAAELG----ADLVVASTHKTGGSLTQSSMLLHNEGLVK-------YHSIKKRVLVFHTTSPSYLLMASLESSRKL : 268
ChvarUNT-a : VPLLVDEAHGAHLGL-------HPALPPSALQQG----ADMAVQSTHKQLSALTQAGMLHVRGGRVA-------RERVSRALQVLQSSSPSYLLMSSLDAAREQ : 273
PhpatUNT-a : IPLIIDEAHGAHFRF-------HSKLPQTALEQG----ADIAVQSTHKVLGSLTQSAMLHAQGSRIN-------RARLAQCLQVLQSSSPSYLLLASLDASRAH : 274
NopunUNT-a : IPLLVDEAHGAHFAF-------HPELPTPALAAG----ADLTVQSIHKVLGAMTQASMLHIQGDRID-------CDRISKALQLVQSTSPSYLLLASLDAARQQ : 270
SyspeUNT-a : CCVLVDEAHGCHFATG-----VDHPLPPSALHCG----ADLVVHSLQKSAAALAQTAVLWLQGERLD-------PVRVERSLGLLQTTSPSALLASCEEALQH : 274
PachrUNT-a : LTVLVDEAHGSYFAV------VPDELPKSALASG----ADLVVGSLHKSAGGISQSAVLWLQHERVK-------ASRVELALYWLQTSSPSAALLASGEAALNY : 269
StcoeUNT-a : LPLIVDEAWGAHLPF-------HPDLPSWAMDAG----ADICVTSIHKMGSGLEQGSVFHLRGDLVP-------PKLLGMRADLLSTTSPSVLLFAGLDGWRRQ : 269
MysmeUNT-a : KPLIVDEAWGAHLPF-------HENLPTWAMNAG----ADVCVVSVHKMGAGFEQGSAFHLQGDLID-------QDRLSACADLLMTTSPNVLVYSAIDGWRRQ : 269
BoperODCcw : DTLHFDEAWLPHAAFHEYYRDMHAIGPDRPRSQD---AMVFATHSTHKLLAGISQASQIIVQESE----SRKLDRNLFNEAYLMHTSTSPQYAIIASCDVAAAM : 299
RalstODCcw : DTLHFDEAWLPHASFHPFYRDMHAIGQGRARSKD---ALVFATQSTHKLLAGLSQASQILVQDSE----TRKLDTYRFNEAYLMHTSTSPQYSIIASCDVAAAM : 299
LebifUNT-w : STLHFDEAWLPHASFHRFYTGMHAIGSDRPRPKE---SMIFATQSTHKLLAGLSQASQILVQNSE----KETLDRNLFNEAFLMHTSTSPQYAIIASCDVAAAM : 298
PseaeLDCcw : EVLHFDEAWYAYAAFHEFYDGRYGMGTSRSE-EG---PLVFATHSTHKMLAAFSQASMIHVQDGG----TRKLDVARFNEAFMMHISTSPQYGIIASLDVASAM : 297
EscoADCi-w : DRLHFDEAWYGYARFNPIYADHYAMRGEPGDHNG---PTVFATHSTHKLLNALSQASYIHVREG-----RGAINFSRFNQAYMMHATTSPLYAICASNDVAVSM : 298
FuvaUNTi-w : DYLHFDEAWYGYAKFHPIYAERFAMRGNPKDYKNK--STVFATHSTHKLLNALSQASFIHVRNG-----NNPIDEDRFNQSYMMHATTSPLYAIAVSNDIGAAM : 284
MebooUNTiw : DSIHFDEAWYGYARFNPLYRDRFAMRDGAKDKKG---PTVFATQSTHKLLAALSQASMVHVRNG-----RVPIEHARFNEGFMMHSSTSPLYTIIASLDVSTKM : 298
StambUNT-w : PRLHFDEAWFAYARFHPLYAGRYGMAVDEETFPGPDRPTIFATQSTHKLLAALSQGAMVHVRPAP----RAPVEHDRFNEALMMHGTTSPLYPMIASLDVATAM : 302
AcspeUNT-w : PRIHFDEAWYAYAKFHEIYRGRFAMGVPDEIPDR---PTIFSVQSTHKMLAAFSMASMVHIKLSQ----RAPLDYDQFNESFMMHGTTSPFYPLIASLDVAAAM : 299
VivadUNTiw : PRIHFDEAWYAYAKFHPIYAERFAMGVSGKIKNM---PTIFAVQSTHKMLPALSMASMIHVKKSD----RAPLDFDDFNDSFMMHGTTSPYYPIIASIDVAVSM : 299
EscoLDCi-w : KSIHFDSAWVPYTNFSPIYEGKCGMSGGRVEGKV-----IYETQSTHKLLAAFSQASMIHVKGD--------VNEETFNEAYMMHTTTSPHYGIVASTETAAAM : 286
EscoLDCc-w : PSIHFDSAWVPYTHFHPIYQGKSGMSGERVAGKV-----IFETQSTHKMLAALSQASLIHIKGE--------YDEEAFNEAFMMHTTTSPSYPIVASVETAAAM : 286
PypisUNT-w : KKIHFDSAWSPYAPFNPIYENKFGMCGEPTAGKT-----IFETQSAHKMLASFAQASYVHVKGE--------YDESVLDEVYMMHTTTSANYPIVASAETGAAM : 286
FratuLDCcw : KKIHFDSAWIPYAIFHPIYKHKSAMQIEPRPEHI-----IFETQSTHKLLAAFSQSSMIHVKGD--------YNEEVLNEAFMMHTSTSPFYPIVASVETAAAM : 287
ElminUNT-w : HFLHFDEAWYAYGHFHPFYANRYAMSPYQKKRGVK-RPPVFATQSTHKLLWAFSQGSMIHYKMGTN---KFELDVQGLKEANLFHASTSPFYPLFASIDISAAI : 302
StlugODCiw : DYILFDSAWVGYEQFIPLMKKSSPLLLLDLDEND----PGIFVVQSTHKQQAGFSQASQIHKKDKHIKGQKRYINHKRFNNAYMQYASTSPFYPMFASLDINARM : 303
StepiODCiw : DYILFDSAWVGYEQFIPLMKKSSPLLLLDLDEND----PGILVVQSTHKQQAGFSQASQIHKKDKHIKGQKRYINHKRFNNAYMQYASTSPFYPLFASLDINAKM : 303
OeoenODCiw : DYILFDSAWVGYEQFIPMMKDSSPLLLLNLGPDD----PGILVTQSTHKQQAGFSQASQIHKKDSHIKGQKRYINHKQFNNAYMKFSSTSPFYPLFATLDINAKM : 303
EscoODCi-w : DYILFDSAWVGYEQFIPMMADCSPLLLDLNEND----PGILVTQSVHKQQAGFSQTSQIHKKDSHIKGQQRYVPHKRMNNAFMMHASTSPFYPLFAALNINAKM : 303
EscoODCc-w : DYILFDSAWVGYEQFIPMMADSSPLLLELNEND----PGIFVTQSVHKQQAGFSQTSQIHKKDNHIRGQARFCPHKRLNNAFMMHASTSPFYPLFAALDVNAKI : 303
DiinvUNTiw : DYIYFDSAWVGYEQFIPMLRDCSPLLLNLGPDD----PGIIVSQSVHKHQAGFSMSSQIHKKDAHIKGQERYLPHKRLNNSFMVNASTSPFYQVFASLDMNARI : 303
VedisUNT-w : DYIFFDSAWVGYEQFIPMMDCSPLLLELGPND-----PGILVTQSVHKQQAGFSQTSQIHKKDKHIKGQDRYVDHKHFNNSFMMHASTSPFYPLFASLDVNAKI : 303
LabacODCiw : DYVLFDCAWGGYEQFVNIMKQLSPLQYQYTADD----PGILVTQSIHKQQAGLAQTSQILKKDAHIKGQKRYVDHKHFNNAYLKYVTTSYSYPIYASLTVNTAL : 303
GrbetUNT-w : DYILFDSAWAGFMKFHPLFRGRFGMGLRNLGEND---PGIIVTQSTHKQLASFSQASQIHKKDSHLKGQDRQIGHNRFNEMFLMHASTSPFYPLFASLDVGAQM : 308
TethUNT-a  : RYLIVDEAHGAIHYEN-------KSDKRTALSSG----ADITVSSMHKSLGSLVATSLINVAADSKFP------TKKILDAYYTFATTSPNILLLASVYDAILE : 270
               D A     f                           S hK      q s                              33Sp y   a

           320          *         340          *         360          *         380          *         400          *
StlugYaaOa : YQQYD-------SRIFFKRRQQFIEMMQ----DKGFIMIQMA-------------------------------------------------------------------- : 287
StaurYaaOa : YKTYD-------STVFFDKRAQLIECLE----KKGFEMLQVD-------------------------------------------------------------------- : 287
LimonYaaOa : VATYT-------AADVEAFWKMRARWIKWLT-KNKFEVILPD-------------------------------------------------------------------- : 298
BasubYaaOa : VQHIIEEQKLSDILQRIETLKQTFDSLTNAEAVNPANPLIIT-------------------------------------------------------------------- : 311
CldifYaaOa : YENKG-------KEIMEELINNIITFKN--KVDSNINIYDTN-------------------------------------------------------------------- : 303
CrserUNT-a : MATNG-------HDLLQRAIDLAEDARRRIN-NIEGLFAPGR-----------------------------------------------------ERAHTEAVYD--- : 313
CoproUNT-a : MATEG-------AELLSSAINVAQYLREAIN-EIPGLQCFGR-----------------------------------------------------ERVGKYGIFD--- : 312
CaexiODC-a : MALHG-------KEMVDKAIEVAEYARERIS-ALDGFKVITD-----------------------------------------------------KTINGKDVYA--- : 313
UTG1RsUNTa : AFLRG-------EENFNRVIKAAEWGRAYINDNINSMKCFTR-----------------------------------------------------QEVQK-LGFD--- : 313
ElubaUNT-a : AVHKG-------EKMLDRVIKTAQRARRRIN-ALEHFSCFTR-----------------------------------------------------EDIKKNNGRD--- : 313
MeoxyUNT-a : MATEG-------EKLLGKAIDLARDARDRIN-RIPGLSCFGE-----------------------------------------------------PDLR---GRDF-- : 311
LeferLDC-a : MATEG-------KKLLSKAIDLARKARERIR-KIPGLTCIDR-----------------------------------------------------DEVKAASGGDN-- : 314
BacihADC-a : LATEG-------YSLIDRSIKLADQLRADIN-KIPFLHCVGK-----------------------------------------------------EIIGSPATFD--- : 313
PesimADC-a : LATVG-------REMIDEAINLAGYIREAVN-EIPYLYCVGS-----------------------------------------------------EILESEAAFS--- : 313
BasubADC-a : LATEG-------RQLAEETIKLANQTRDRLN-QIEGIYCVGS-----------------------------------------------------EILGSKAAYS--- : 313
FunucODC-a : IATQG-------QELLTRTIELAKYFREAN-RIPGIYCFGE-----------------------------------------------------ELVGKDGFFA--- : 314
FuulcUNT-a : IATEG-------YELLDRTIKLAKRFNEVN-RIPGMFCFGD-----------------------------------------------------EIVGREGIYA--- : 314
IlpolUNT-a : IATEG-------RELLTKSISLAHKLRSEIN-KIPGVSSFGI-----------------------------------------------------EIVGREGIYA--- : 314
FunecODC-a : IATQG-------RELLTKAISLAHYFREEAN-RIPGIYCFGE-----------------------------------------------------EIIGREGAFA--- : 314
ClbotODC-a : IALEG-------KELLDKAIDLATYARDEIN-KIPGFYCFGK-----------------------------------------------------ELLGKPGVYA--- : 313
CltetUNT-a : IALNG-------KELLDKSIALSNYAREEIN-KIPGFYCFGK-----------------------------------------------------EIVGRPGVFA--- : 313
ThethUNT-a : MATEG-------RELLDKTIELAEYARKEIN-NIKGLYCFGE-----------------------------------------------------EIVGRDGAYD--- : 313
ThtheOLDCa : MVTEG-------KQLLDKTIELAEYARKEIN-NINGLYCFGN-----------------------------------------------------EIVGRDGAYD--- : 313
LebucUNT-a : LAING-------RELFEKTVKFAEYARNEIN-KLGGYYAYGK-----------------------------------------------------ELIDGDSVYD--- : 311
BrmurUNT-a : LAING-------KELFKTVINMADYARKEVN-KIGDYYAFGE-----------------------------------------------------EIINNDDIYS--- : 311
StpneADC-a : LALRG-------KESFEEVIELSEYARHEIN-AIGGYYAYSK-----------------------------------------------------ELIDGVSVCD--- : 311
SerumADC-a : LALHG-------RESFEKVSSMAEYARAEIN-EIGGFYAYGR-----------------------------------------------------ELIDGDSVFD--- : 311
HutheADC-a : LATKG-------SDVLEETIRLARMAREEIN-KIEGLYAFGK-----------------------------------------------------ELIGTPGCHD--- : 312
PsychADC-a : LVTQG-------DEIFNDLIEMISKFREELN-SIPGIRCFKS-----------------------------------------------------ILS--DKLYD--- : 309
CldifADC-a : LVENG-------EKQLSKAINLSRYAKSKIN-KIDGIKVLST-----------------------------------------------------EILKQKGVKF--- : 312
AclaiUNT-a : IAVHG-------KEALDKVIEMTRKTRDEIN-QISGLKAMTK-----------------------------------------------------QDVINNKGFN--- : 312
MeblaADC-a : LATQG-------EEIFHELVHECRKVKKEIS-KIPGLSVLDE-----------------------------------------------------NEGVTT--MT--- : 310
ChvarUNT-a : ASTP---------AAHEEAIAAAAYARQQLERLPGVQLLSAG------------------------------------------------------QVAGSGGVG--- : 315
PhpatUNT-a : MEVNRQ------DSKLDAAISLAFMVRNAIQQLPGLMILDLAS-----------------------------MPSD-------------AVAESSIAG--- : 324
NopunUNT-a : MVLHG-------KMLMSRTIQLANEARTIISQIPGLSVLQILS-----------------------------PPYQGGLGGSSSLGGLVGSPGFVA--- : 330
SyspeUNT-a : WQRRKG------RRQLLRRIQEAAALAEDIRNS-------------------------------------------------------------GVPLLNN--- : 308
PachrUNT-a : LASEPG------YRNLKRSVKLGHQLRTDISCA-------------------------------------------------------------GFPLVVN--- : 303
StcoeUNT-a : MALGG-------QEIMGGAIELAAGVRAAVEEIDGMHVNDR-----------------------------------------------------EDFCGPGLAHG--- : 314
MysmeUNT-a : MVEHG-------HRIFGAAIDLADDTRRIEELLDDVEVLD------------------------------------------------------EELLGAQASHD--- : 313
BoperODCcw : MEPPG------GTALVEESIREAMDFRRAMRKVASEFG--------KDDWWFKVWGPNRLAPEGIGHR-----------------DDWVLESGDHWHGFG--D : 369
RalstODCcw : MEAPG------GTALVEESIAEALDFRRAMRKVEQEYVGTNGGNGRGDDWWFKVWGPNDLSDEGIEER-----------------EAWMLKANERWHGFG--D : 377
LebifUNT-w : MESPG------GNALVEESIEEALDFRRAMRKVGLELE---------EDWWFSVWGPEALADEGAGER-----------------DEWILKANDRWHGFG--D : 367
PseaeLDCcw : MEGPA------GRSLIQETFDEALSFRRALANVRQNLD--------RNDWWFGVWQPEQVEGTDQVGT-----------------HDWVLEPSADWHGFG--D : 367
EscoADCi-w : MDGNS------GLSLTQEVIDEAVDFRQAARLYKEF-------TADGSWFFKPWNKEVVTDPQTGKTY---DFADAPTKLLTTVQDCWVMHPGESWHGFK--D : 384
FuvaUNTi-w : MDGNS------GKSLIKEVVKEAVEFRQEIGKLYKEF-------TAKNDWFFKPWNTEKVIDPKTKKEY---NFEEAPMELLTTEQDCWIMHPEDKWHGFA--D : 370
MebooUNTiw : MDGAS------GRLITTESIEEAIRFRRTMARIEKEIGTG----KTRTDWWFPMWQPDTVTDPKTKKKI---AFATAPLETLRDNPSCWVLHPKDAWHGFE--D : 387
```

```
StambUNT-w : MDGPQ------GQWLIDEAISEAVRFRQEMVRIGRRIEAA----GDRPPWFFGVWQPDEVTDPASGARL---PFEEAPADLLRTEASCWHLDPDADWHGFP--G : 391
AcspeUNT-w : MDEPA------GPTLMSETLQDAISFRKAMSSVAHRLRA-----AEQG-WFFRLYQPEYVFDPLDGETY---LFEEAADGLLTNRSSCWTLKPGEDWHGYQDED : 388
VivadUNTiw : MKGSS------GTHLVQESIEDAIAFRKAVVSIKRKIKE-----KNPADWFFDIMQPVEVKDPKTGRKY---SFEDAPVSLLSREPSCWALDPNAKWHGFARED : 389
EscoLDCi-w : MKGNA------GKRLINGSIERAIKFRKEIKRLRTES----------DGWFFDVWQPDHIDTT-----------------------ECWPLRSDSTWHGFK--N : 349
EscoLDCc-w : LRGNP------GKRLINRSVERALHFRKEVQRLREES----------DGWFFDIWQPPQVDEA-----------------------ECWPVAPGEQWHGFN--D : 349
PypisUNT-w : MTGNQ------GRRLLQNSIDRAMTFRRELARLYDES----------DTWFFKCWQPDDISET-----------------------KCWPISRGERWHGFL--G : 349
FratuLDCcw : MEGEQ------GYNLIDKTINLAIDFRRELIKLRSEA----------NGWFFDVWQPDNISNK-----------------------EAWLLRNGDKWHGFK--N : 350
ElminUNT-w : MDSD-------GEKLMGKALDEAIKFRKEIINRYKKHKK-------ADSWYFELWQQEMKLKHVNKDP-----------------KNWIIDGSEPWHGFEG-- : 372
StlugODCiw : QEGPM------GEKLWNDCLTVSTNARKEILAKCK---------------YIKPFLPEKVN---------GKKWSDAPTEEIISNLDYWKFNPEESWHGFNG-- : 375
StepiODCiw : QEGEL------GEKLWNDCLTISTNARKKILNRCK---------------YIKPFIPEKVQ---------GKDWEEANTKEIISNLDYWKFNPDEKWHGFKG-- : 375
OeoenODCiw : QEGEA------GKKLWHDALVTSVNARKNLLKNAT---------------MIKPFLPPVVH---------GKPWQDADTEKIVSDIDYWKFEKGAKWHGFDG-- : 375
EscoODCi-w : HEGVS------GRNMWMDCVVNGINARKLILDNCQ---------------HIRPFVPELVD---------GKPWQSYETAQIAVDLRFFQFVPGEHWHSFEG-- : 375
EscoODCc-w : HEGES------GRRLWAECVEIGIEARKAILARCK---------------LFRPFIPPVVD---------GKLWQDYPTSVLASDRRFFSFEPGAKWHGFEG-- : 375
DiinvUNTiw : QEGEG------GALLWKECMELSVEARKAVIRNCK---------------YLKPLVPPVVH---------GKNWEEWDTEEIINDIAYFTFEPGGKWHSFQG-- : 375
VedisUNT-w : HEGEL------GQTLWRECVEVAIDARKAVLKQCK---------------YLRPLVPPIVH---------GKPWEEGNTQEMACDVKYFAFEPEAKWHSFNG-- : 375
LabacODCiw : AKGEA------PKKWWEEAMRKGIRFRKAINQKSK---------------LFKTLNAQEIN---------EK-----SEQELMDNLSYWKMEETDDWHGFKG-- : 370
GrbetUNT-w : MKGRS------GEVLWDDTVRLGIELRKKIRLTRKEYKQAQ--TSPDLQWFFEPFVPKRVQPSEKAEAFAGLRWEDAPTDLLASDPEQWMLRPGDDWHGFPD-- : 402
TethUNT-a  : LSENG-------EEYLQNAIQRNNQFRNEIEKLDNIIIDSFD---------------------------------------------------------RSQK--- : 309

                420         *       440         *       460         *       480         *       500         *       520
StlugYaaOa : ---------DPLKLTVQ--HKHMLG----------YQIQDWFE-KMSVFVELADDYQVLLILPLWHEQ--DKYPFDELIERIR-MQPVEDLINHNQQS------- : 360
StaurYaaOa : ---------DPLKLLIK--YEGFTG----------HDIQNWFM-NAHIYLELADDYQVLAVLPLWHHD--DTYLFDSLLRKIEDMILPKKSVSKVKQ-------- : 360
LimonYaaOa : ---------DPLKIIVR--KTGYTG----------YELQAIFE-ESSYFPELADESQVLLILPLIKKG--IDFTP---ISRIH--SPMKKEIAKEPY-------- : 366
BasubYaaOa : ---------DPLKLTIRS-KRGHSG----------YTLQSILE-RANIFTELADENQVLLVLPLGGKRRINAEIIRSIDEEIEKTPPDQTFVSAEWG-------- : 387
CldifYaaOa : ---------DKTKIFISSKNMGLTG----------YELENILRNKYNIQVELSNYYGVLLICTIGNTAEDFSSLETALNNVVKEDFKFEKLENKKYP-------- : 381
CrserUNT-a : --------VDLTKLTVHVSRLGLTG----------FQVEEILNRDYNVEIELSDLENILAFITIGTPREAVEILIKALEDISRKRHGKTS--TIA---------- : 388
CoproUNT-a : --------VDLTKITITVLGLGLTG----------YKVEQLLNDELGVEVELSDPNNILAFVTIGDDYDNADKLIKAMSNLSERFFGKLPPLETA---------- : 389
CaexiODC-a : --------FDPIKLTINVQELGYSG----------FEFEHILNKN-GIEIELADLQNVLLFITIGTSKRDIDTLVSVLKRIEPR---KEK--STV---------- : 384
UTG1RsUNTa : --------LDVTKLTVNVTKTGLSG----------YEIESILAKEYNVQLDYADLFNLVAIMSEGSEKSDVEVFVNALENISEKYRGKQKN---W---------- : 387
ElubaUNT-a : --------LDVTKLTINVTRAGMSG----------YDVEDILAKEYNIQVDCADTFNLIAIMGIGSTPRDTEALVSALADIEKKIAASVVGPAPD---------- : 390
MeoxyUNT-a : -------FLDVTKLTICVKELGRSG----------FEVSATLNAEFGIQVEMADLFNILVIVSIGDRRDDLDRLVAALGIVAGRGTR-PQDPRAI---------- : 388
LeferLDC-a : -------DLDETKLAIGVKGIGLTG----------YQVSQKLNHEFGIQVEMADLHHVLVIVSIGDHREDLDRLVRAMEAISHQAMT-LKSIIPI---------- : 391
BacihADC-a : --------YDPTKLIISVKDLGISG----------YEVEKWLREKYNIEVELSDLYNILCIITNGDTKQEANILIQALQDLSNEFKHLAANGNKH---------- : 390
PesimADC-a : --------YDPTKLIISIKELNITG----------YDVEKWLREKHNIEVELSDLYNILCLITPGDTKTEADLLIAALKDLADEFKEMAAEHEK----------- : 389
BasubADC-a : --------YDPTKLIISVKSLGLTG----------HDVEKWLRESFNIEVELSDLYNILCIFTPGDSQNDADRLVEALTEIAQQMSEQDVTHQQ----------- : 389
FunucODC-a : --------FDPTKITISAKELGLKG----------GELESLLVDDYNIQMELSDYYNTLGLITIGDTEESVNKLLDALRDISRRFFGKGKTLEK----------- : 390
FuulcUNT-a : --------FDPTKVTVTAKELGLTG----------FELETLLVDDYNIQVELSDFYNVLGLITIGDSDESIDKFIAALKSISERFYGKVTKVKR----------- : 390
IlpolUNT-a : --------FDPTKLTITARDLGLTG----------FQLETLLVNEYNIQVELSDYYNVLAIITLGDSEESIGKLSDALRDISERFFGK-EEIHR----------- : 389
FunecODC-a : --------FDPTKITFTAKELGFTG----------TELEDMLTADYHIQMELADFYHTLGLITIGDSKESIDRLIAALRDISNRFSNQGRKLTH----------- : 390
ClbotODC-a : --------LDPTKITINCRDLGITG----------YDLDMLLCEKYHIQVELSDLYNVLAVGSFGDTQESIDALINALKEISNEHYGKGTKQN------------ : 388
CltetUNT-a : --------IDPTKITITCRDLGITG----------FELDMILSNQYHIQLELSDLYNGLAVGSFGDTKENIDALINALKEISKEYVNNENKKS------------ : 388
ThethUNT-a : --------FDPTKVTITAKGLGISG----------HQLEKILAERYYIQPELSDMYNVLCVFSIGDTKEKVDYLIRALREISDELYNENIEIA------------ : 388
ThtheOLDCa : --------FDPTKITITAKGLGITG----------HELENILAKKYYIQPELADMYNALCVFSIGDTKENVDYLIRALREISETLYKDNVEMS------------ : 388
LebucUNT-a : --------FDTTKLSVYTKDIGLAG----------IEVYDILRDDYEIQIEFGDLGNILAIISAGDRGLEIERLISSLAEIK---RLYSKDSTG----------- : 384
BrmurUNT-a : --------FDNTKLCIHTRKIGLAG----------IEVYDKLRDEYNIQIEFGDIGNVLAIISVGDRNLELERLIAALAELK---RKYKKDYTH----------- : 384
StpneADC-a : --------FDVTKLSVYTQGIGLTG----------IEVYDLLRDEYDIQIEFGDIGNILAYISIGDRIQDIERLVGALADIK---RLYSRDGKD----------- : 384
SerumADC-a : --------FDVTKLSVFTQGIGMTG----------IEVYDMLRDEYDIQIEFGDIGNILAYISIGDRIRDIERLVGALEDIAD--RSAAADRKE----------- : 385
HutheADC-a : --------FDETKLGICVSGLGYTG----------YEMEAKLRKEYNIQIEMSDLSNILAIVSIGDREENLVALINALKDIA---AKTEKKEYP----------- : 385
PsychADC-a : --------FDDTKIGINLHGLGLTG----------FEGYDILREKYNIQMELADTHNILGIASIGDTWEDYQKLIDALKEMA---ENYEGNNQL----------- : 382
CldifADC-a : --------IDETKLCINVKELNLTG----------FEVYDLLYKNFSIQVELGDSYNILALVSIGTNKSDIDRLVKALSIIAK--VYRKESTLN----------- : 386
AclaiUNT-a : --------YDETKIMVKVSDLGITG----------FEAYNILSDKFGIQMELAETHLILAVLSIGTTQNDLDRFVEGLKGL----VAYKKDVKI----------- : 384
MeblaADC-a : --------YDPTKIVINVTKLGLDG----------FDVYDIMFEKYGIQMELAEPTIVLAVIGIGDTKETIWRLVDAFRDLSDTYYGKYEMLDI----------- : 386
ChvarUNT-a : --------LDPLRLTISLQQLGLTG----------YQAAAVLEQQCGIVAELATPTCVVLAMGIGSTQQHAQALVAAVQQLCQQHYGDNSSSSSSSASSSS---- : 398
PhpatUNT-a : --------TDPLRITVGLWGLGLTG----------FEADDILRFEFGVIAELPSLRSITFAVSVGSSHRDATRLIDSFTALSSR-YACRTDQIVGPISNPL---- : 406
NopunUNT-a : --------LDKTRLTVTVSGLGLTG----------FEADEILDEKFAVTAEFASLQHLTFIISLGNTPADIKQLVQGFTILAKEYRATNLNVTNPNLQN------ : 411
SyspeUNT-a : --------QDPLRLLLHTGQAGITG----------LDADDWFLPRG-LVGELPEPASLTFCLGLARHRGLGRQMRHHWQNLLRSFPDRAPLPA------------ : 382
PachrUNT-a : --------QDPLRLVLHTAGLGITG----------LEADSWLMAKG-LIAELPEPGCLTFSLGLSPDKRLAKVMLPILENFQEQLLG-LPLSP------------ : 376
StcoeUNT-a : --------FDPLPVVIDLEGLGVSG----------FRAADWLREHRGVVAHLTDHRRIGAQITHGDDRGTAGELLTALKDLALAAPGLGSAPG------------ : 389
MysmeUNT-a : --------LDRLQILIDVSATGTSG----------YQAADWLRENCQVDVGMSDHRRILATMSVADGSDTAGRLLHALSLWRKAADDFAPPPP------------ : 388
BoperODCcw : LAPGFNMLDPIKATIITPGLDMSG--SFGESGIPAALVSKYLAEHGVVVEKTGLYSFFILFTIGITKGRWNTLLTALQQFKDDYDRNQPLWRILPEFCRG-HRR : 470
RalstODCcw : LAEDFNLLDPIKATIITPGLDVDG--KFSESGIPAAIVTKYLAEHGIIVEKTGLYSFFIMFTIGITKGRWNSMVTELQQFKDDYDNNQPLWRVLPEFVRQ-YPQ : 478
LebifUNT-w : IAEGFNMLDPIKATVITPGMSVEG--EFADWGIPALILTKYLAEHGIIVEKTGLYSFFIMFTIGITKGRWNTMVTELQQFKDDYDSNQPLWRVMPKFTVA-YPK : 468
PseaeLDCcw : IAEDYVLLDPIKVTLTTPGLSAGG--KLSEQGIPAAIVSRFLWERGLVVEKTGLYSFLVLFSMGITKGKWSTLVTELLEFKRCYDANLPLLDVLPSVAQAGGKR : 469
EscoADCi-w : IPDNWSMLDPIKVSILAPGMGEDG--ELEETGVPAALVTAWLGRHGIVPTRTTDFQIMFLFSMGVTRGKWGTLVNTLCSFKRHYDANTPLAQVMPELVEQYPDT : 486
FuvaUNTi-w : LPENWAMLDPIKVSILAPGMGDDG--ELLEKGVPAALVSAYLAKYGIVPTRTTDFQVMFLFSMGITEGKWSTLVNVLLSFKKHYDNNTLLKDVLPELVAAHPEH : 472
MebooUNTiw : LPDNYCMLDPIKVTVLTPGVKEDG--SLAAWGIPAAIVVKFLDTRGIINEKSGDYSILFLFSMGITKGKWGTLITELFEFKRRYDENAPLDEVFPDLVAQWPDR : 489
StambUNT-w : LTDGYSMLDPIKVTLTCPGLDASG--VMSDWGVPARVLTAYLTTRGIVVEKTDGYTTLVLFSMGITKGKWGTLLDALMDFKDHYDSDVPLDRVLPALVAEHPRR : 493
AcspeUNT-w : IADDYCMLDPSKVTIILTPGVNAQG--VVSDWGIPAAILTEFLDGRRVEIARTGDYTVLVLFSVGTSKGKWGALLENLFEFKRLYDSEAPLEEALPELVLKYPAR : 490
VivadUNTiw : IADTDCMLDPVKVTITCPGITPDG--KYQKTGIPGYIMTKFLDDRRTEIARTGDYTLLIILFSVGTTKGKWGTLIESLLEFKRLYDNGALVADAIPSLAEISPEY : 491
EscoLDCi-w : IDNEHMYLDPIKVTLLTPGMEKDG--TMSDFGIPASIVAKYLDEHGIVVEKTGPYNLLFLFSIGIDKTKALSLLRALTDFKRAFDLNLRVKNMLPSLYREDPEF : 451
EscoLDCc-w : ADADHMFLDPVKVTILTPGMDEQG--NMSEEGIPAALVAKFLDERGIVVEKTGPYNLLFLFSIGIDKTKAMGLLRGLTEFKRSYDLNLRIKNMLPDLYAEDPDF : 451
PypisUNT-w : ADEDFNYLDPIRVSVLTPGMDPTG--QLMEEGIPAAVVSRYLNNHGVVTEKTGPYHMLFLFALGVDELRTKALLRALQDFKRDYDDDVPIREAMPDLFKLDPVF : 451
FratuLDCcw : VDGDFLSLDPIKITILTPGIK-DN--DVQDWGVPADVVAKFLDEHDIVVEKSGPYSLLFIFSLGTTKAKSVRLISVLNKFKQMYDENTLVEKMLPTLYAEDPKF : 451
ElminUNT-w : LEKENVLLDPLKVTLLTPGIEENG--EMQPFGIPASIVSKFLNTRKIIPEKTGFYNILFLFAPGVGEQKEERLVKALDEFKTLYDRNEPISGIFPELINDYPHI : 474
StlugODCiw : YGKDQYFVDPNKFLITTPGINPED-DSYMDFGIPAVILANYLRENGIIPEKNDLNSILFLMTPASDEAKMNNLIAKLIKFESLIDQDAPLDKVLPRLYHQNFER : 478
StepiODCiw : YGNEQYYIDPNKFLITTPGIDSKD-DSYMDFGIPAVILANYLRENGIIPEKNDLNSILFLMTPAEDTAKMNNLIAKLIQFERYIDQDAPLEVVLPRLYYNNYER : 478
OeoenODCiw : YADNQYFVDPNKFMITTPGIDVET-GEYEDFGIPAVILANYLREHGIIPEKNDLNSILFLMTPAETQAKMDNLVTQIVKFESLVKADAPLDEVLPRLYSEHQDR : 478
EscoODCi-w : YAENQYFVDPCKLLITTPGIDARN-GEYEAFGVPATILANFLRENGVVPEKCDLNSILFLLTPAEDMAKLQQLVALLVRFEKLLESDAPLAEVLPSIYKQHEER : 478
EscoODCc-w : YAADQYFVDPCKLLITTPGIDAET-GEYSDFGVPATILAHYLRENGIVPEKCDLNSILFLLTPAESHEKLAQLVAMLAQFEQHIEDDSPLVEVLPSVYNKYPVR : 478
DiinvUNTiw : YGKGQYFIDPMKLLFTTPGINVET-GRYEKFGIPGIVLANYLRENAVIPEKCDLNDILFIITPAETKAKINNLISRILHFEAFVDNDAPMAKVLPNIYHTYQDK : 478
VedisUNT-w : YGEGQYFIDPCKFQLITPGIDVET-GEYEEFGIPANILANYLRENRIIPEKCDLNTILFLMTPAESKHKMDALVAELVRFEELIDRDVPMEEVLPSIYYSHIDK : 478
LabacODCiw : IAKDEAIISPLKLTVVCPGINLAT-GKYEETGIPGKVIGEYLTEKRVITCKSDLYSTLFLLTPGERDADLEALLTSFLEFEEYYLRDAPLEQVLPRLVKQNPER : 473
GrbetUNT-w : LHPGWVMTDPNKLTLLTPGFDGRNEDNYADHGVPAPVVAEYLRQNRIVPEKNDLNSLLFLLTPGIESSKAGTLLSYLVTFKNLHDQNVLLSDAIPDFVARRRER : 506
TethUNT-a  : --------DQLKTIFGIKNVHG-----------YQLAKILQDKYNIWVEKYNSISVIVTIHINTEDADIEKLIKAVREISKKYEHKTQYDQSIN--------- : 384
                    d            g                   e

                  *       540         *       560         *       580         *       600         *       620
StlugYaaOa : ----------------------------SYTRLPCAAG---SYETMDIT---HVTWVGLDNAQGMILAQHITPYPPGIPLFFTGERIT---QNMIKLIANYI- : 425
StaurYaaOa : ------------------------------TQLLTTEG---NYKPKRFE---YVTWCDLKKAKGKVLARHIVPYPPGIPIIFKGETIT---ENMIELVNEYL- : 423
LimonYaaOa : -----------------------------EMSTPCASGLALTYEEMHAR---ATEFVLLDEATTRVSAETISLYPPGIPAIIRGEGIT---EKHIRELKNIR- : 433
BasubYaaOa : ------------------------------VQPVTVLP--YPKKVLHSF---KKEYVSFEEAAGRINAEDIIPYPPGIPMIMAGERIT---KESVQKLSRLIS : 452
CldifYaaOa : ---------------------------------VVIPKKILTPREAFYK---TKKSVKIYDSIGKVCGECIVPYPPGISVISPGEVIS---KEIIDYLMFCHS : 445
CrserUNT-a : ---------------------------PPPPIPNIPEMVLFPFEAYYA----KRERVRMEDSVGRIIAELVTPYPPGIPITVPGEVMT---HECVDYMVFMRD : 457
```

```
CoproUNT-a : ----------------------------AVHTFPVLPEVVLTPREAFFA----EKKVVPFREAVCAISAESVVLYPPGIPVIAPGERIT---EESFDYVMKRKE : 458
CaexiODC-a : ----------------------------RMPKLPEAPSFAMLPFEAFQR----EFEVVPIRETKGRVSWGIVAPYPPGIPVIAPGMIID---EECIEFTQEVFE : 453
UTG1RsUNTa : ----------------------------ILKIPSLATEMVMRPREVFLSN---STKKVSIKKAAGHVAAQTITPYPPGIPVVIPGERIT---KEICDYIIDMSS : 457
ElubaUNT-a : ----------------------------MTDLPPLTTEMVMAPREVFFSK---KIKRIPLSRAAGHISAQTITPYPPGIPVLIPGERIT---SQVVDYVQELAR : 460
MeoxyUNT-a : ----------------------------PFLLPPLGQQCHLPPRDAFFA----PSEYLPLAKAEGRISADIITIYPPGVPVIVPGEEVG---SSAIDYIRSLAS : 457
LeferLDC-a : ----------------------------NVKKPPYAIASSRMNPREAFFS----SHQSVPIEEALGKTAADIVTVYPPGIPLLIPGEEIT---QTVLDHIILMRE : 460
BacihADC-a : ----------------------------IEVLLPTIPVLALSPRDAFYS----DTEVVAIDESVGRIIAEFIMVYPPGIPIFIPGEIIT---QENIDYIKANME : 459
PesimADC-a : ----------------------------IEVLLPDTPPLALSPREAFYA----ETEVVPIEESVGKISAEFIMVYPPGIPIFIPGELIT---EDNINYIRKNIE : 458
BasubADC-a : ----------------------------TEVLLPEIPLLAMTPRDAFYA----NTEVIPLKEASGRIIAEFVMVYPPGIPIFIPGEIIT---EENISYIFKNLD : 458
FunucODC-a : ----------------------------NIIKLPETPELVLMPREAFYS----EKNKVPFKESVGKISGEMIMAYPPGIPIIIAGERIS---QDIIDHIEELKE : 459
FuulcUNT-a : ----------------------------NFIKMPSVPEQVLIPREAFYS----EKNTILFEESACKICGEMIMAYPPGIPIITPGERIT---DEIIDYIKDLKE : 459
IlpolUNT-a : ----------------------------KSLKMPAIPEPVLIPREAFNS----VKNKILFAESECKICGELIMAYPPGIPIICPGERIT---SDIIEHIKELKD : 458
FunecODC-a : ----------------------------QLLKMPEIPEQVLIPREAFYR----TKTKISFEESIGKICGELIMAYPPGIPIICPGERIT---REILDYIKDMKS : 459
ClbotODC-a : ----------------------------SFIDVPEIPENTAAPRKAFNG----VKECVLLKDSVGKVSGEFIMAYPPGIPVICPGEIIT---REIVDYVERLKE : 457
CltetUNT-a : ----------------------------DFIDIPAIPKQVQIPRDAFNS----EKVVLPLKDSEGRVSGEFIMAYPPGIPVICPGEIIT---QEIIDYIQKLKD : 457
ThethUNT-a : ----------------------------KPIDIPEIPEQVVSPRYAFGS----STVALPLRESVGQISAEFIMAYPPGIPVICPGERIT---LKIIEYVDKMKE : 457
ThtheOLDCa : ----------------------------KALDIPEIPEMVLSPRYAFEC----SNVAIPLEESIGQISSEFIMAYPPGIPIICPGERIT---REIIEYVIEMKN : 457
LebucUNT-a : ----------------------------MFDHEYINPDVVLPPQKAFYS----EKEMIPIKESACKISGEFVMAYPPGIPIIAPGERIT---EEIISYIEYAKE : 453
BrmurUNT-a : ----------------------------LFDHEYINPIVKYSPQKAFYS----NKHSVKLDNSICKTSAEFVMAYPPGIPIIAPGEEIT---QEIITYIKYAKE : 453
StpneADC-a : ----------------------------LIAGEYIQPELVLSPQEAFYS----ERKSLTIDESVGQVCGEFVMCYPPGIPIIAPGERIT---REIVDYIQFAKE : 453
SerumADC-a : ----------------------------LYCGEFIAPELVMSPREAFYA----QGEKLPIEKTVGRVAGEMIMCYPPGIPIITPGERIT---QEIPDYIEYARE : 454
HutheADC-a : ----------------------------KPPIIPPTPKMIVSPRDAFYS----PKKIVPLDKSVGEISGEMIMAYPPGIPVVCMGERIT---QDIVDYIKILKE : 454
PsychADC-a : ----------------------------IDFNELESLELVVSPRDAFYS----ETESIPLSEAICKVSGESIMVYPPGIPLIIQGERIT---KDLIDHIYFLKE : 451
CldifADC-a : ----------------------------EFNMVQINPIIKLNPREAFYA----PKESVEINSCIDRICGESIMAYPPGIPIIAPGELIT---EEIMEYIIFLKN : 455
AclaiUNT-a : ----------------------------AHKIRFTYPDSYTRPREAYHA----PKKYVKIEDALNEVAAENIMVYPPGIPLVIPGEIIN---QEILEDIEFYTA : 453
MeblaADC-a : ----------------------------PEEIVYVPPQILS-PRQAFYA----DKVSVKIGEAVGRVAAEAVMIYPPGIPICIPGELIT---AETIEEILYCQE : 454
ChvarUNT-a : ---------------------------PAAPPAVPSTPDVRLSPRDAFFA----AAEAVPAGQAGGRISAELICPYPPGVPAIYPGEVIT---EVVLQQVQAVLR : 469
PhpatUNT-a : ---------------------------LQDQAIVWENQLMYLSPREAFYS----KSEEIMVECALGRVSADLICPYPPGIAVITPGEVIT---KEAVDYIQAVIA : 477
NopunUNT-a : -------------------------------LLTTQGHTLHFSPREAFFA----LSETLPLAQTSDRICAEIICPYPPGIPVIMPGEIIT---KPVLDYIQQIQG : 478
SyspeUNT-a : ---------------------------FEAPPLPLVTTTAQPPSQAWTA----AHHQVALKDAEGCIAAELICPYPPGIPLIIPGERLD---RQRQCWIERQQL : 452
PachrUNT-a : ----------------------------FMIPPFPDTIELELCADIAWCP----KKEVISLEVATGRIATETICPYPPGIPIIYPGERID---AKRTLWIQQQHS : 446
StcoeUNT-a : ---------------------------VEVPSPAELRMPQVCLPRDAFFG----PTEDVPLDRAAGRVAAEMITPYPPGIPAVLPGERLA---EPVLRYIRSGLE : 460
MysmeUNT-a : ---------------------------IDLPSPAELQLTTKTSPRDAFFA----EVEQIPVEHAVGRIAAEQITPYPPGIPAVVPGERLN---EAVLDYIVTGVQ : 459
BoperODCcw : YER-MGLRDLCQQIHEAYRERDVARLTTEMYLS--DMVPALKPSDAFARMAHREVERVEIDQLEGRVTGVLITPYPPGIPLIIPGERFN---RTIVQYIQFARE : 568
RalstODCcw : YER-IGLRELCDAIHSVYKANDVARVTTEMYLS--NMEPAMKPSDAWAKMAHRETERVAIDDLEGRITAILITPYPPGIPLLIPGERFN---RTIVQYIQFARE : 576
LebifUNT-w : YDR-IGLRDLCQSMHEVYRANNISHLTTEMYLS--PMIPAMKPSEAFAKMAHRDIERVPIDELEGRITSVLITPYPPGIPLLIPGEKFN---TTIIRYIQFARE : 566
PseaeLDCcw : YNG-VGLRDLSDAMHASYRDNATAKAMKRMYTV--LPEVAMRPSEAYDKLVRGEVEAVPIARLEGRIAAVMLVPYPPGIPLIMPGERFTEATRSILDYIEFART : 570
EscoADCi-w : YAN-MGIHDLGDTMFAWLKENNPGARLNEAYSG--LPVAEVTPREAYNAIVDNNVELVSIENLPGRIAANSVIPYPPGIPMILSGENFGDKNSPQVSYIRSLQS : 587
FuvaUNTi-w : YGE-MGFHDLGDEMFAYLKANNPGKVLNEAYST--LPKSEMLPREAYNKIVSQEVELVPADKLVGRVTANSVIPYPPGIPMIMSGENFGDENSPQIKYIKALSL : 573
MebooUNTiw : YGG-MTLPELAAQMHAFKRDHNMCGLLQKAFSA--LPEPVITYAESYKRLVRDEVEQIPVAQAANRIVATGVVPYPPGIPLIAPGEKTGPKTGPVLQYILSLQD : 590
StambUNT-w : YAG-RSLRDLCQEMHTHLRDARLVELLDTAFQQ--LPTPVASPQLCYQRLIRGGTERIPLADAAGRVAAAMVTVTPPGIPVIMPGEDIGEEDGPLLRYIKALES : 594
AcspeUNT-w : YRN-VTLKELSDEMHMVMQQLNLSGLVNAACDE--DFDPVLTPAQTYQKLLRGETEKIKFSEMAGRIAAVMIVPYPPGIPMSMPGERLGGPESPVIRLIMAMEE : 591
VivadUNTiw : AR--MTLKELCQSMHDMMSRLGLIDHLDKAANT--DPEAVLSPAEAYQKVVRYKVEHVRLKDYPSRIAAGMIVPYPPGIPLIMPGERISGDDKGIIGYIEALQE : 591
EscoLDCi-w : YEN-MRIQELAQNIHKLIVHHNLPDLMYRAFEV--LPTMVMTPYAAFQKELHGMTEEVYLDEMVGRINANMILPYPPGVPLVMPGEMITEESRPVLEFLQMLCE : 552
EscoLDCc-w : YRN-MRIQDLAQGIHKLIRKHDLPGLMLRAFDT--LPEMIMTPHQAWQRQIKGEVETIALEQLVGRVSANMILPYPPGVPLIMPGEMLTKESRTVLDFLLMLCS : 552
PypisUNT-w : YMR-MSLQQLTRGLHRVMRKRDLPKLMYHAYDD--LPEMEYTPYQAFQKNLRGETHEVPLAELLGQVSADMILPYPPGVPLVMPGEKVTEKSAAVLDYINMLCE : 552
FratuLDCcw : YED-MRIQEVSERLHQYMKEANLPNLMYHAFNV--LPEQQLNPHRAFQKLLKGKVKKVPIVELYEHTSAVMILPYPPGIPVIFPGEKITEESKSVIILDFILMLEK : 552
ElminUNT-w : YTPEMGLKDLANKVHDFLYINQIFEIIPKMYND--LPEQVMAPHKAYYGLVDSKAEYLSIDEIAGRVAVFMILPYPPGIPLIMPGERFP-QSGGILDFIRMSER : 575
StlugODCiw : YQG-YTIRQLCQELHDFYKEHNTKEFQKKMFLKDYLPEQSMKPYDANIQLLKNNAKLVELDNIVGEIALEGALPYPPGIFCVVPGEKWS---ETAKSYFKILEE : 578
StepiODCiw : YQN-YTIRELCQELHDFYKEHDTKEFQKKMFLKDYLPEYKMKPYDANIELLKNNAKLVELDNIVGEIALEGALPYPPGIFCVVPGEAWS---ETAKTYFKILEE : 578
OeoenODCiw : YEG-YTIKQLCQEVHDFYKNNNTKEYQKEMFLGKYFPEQAMTPYQANVELLKNNAKLVPITDIEGLAALEGALPYPPGIFCIVPGEKWT---KVAQKYFLILEE : 578
EscoODCi-w : YAG-YTLRQLCQEMHDLYARHNVKQLQKEMFRKEHFPRVSMNPQEANYAYLRGEVELVRLPDAEGRIAAEGALPYPPGVLCVVPGEIWG---GAVLRYFSALEE : 578
EscoODCc-w : YRD-YTLRQLCQEMHDLYVSFDVKDLQKAMFRQQSFPSVVMNPQDAHSAYIRGDVELVRIRDAEGRIAAEGALPYPPGVLCVVPGEVWG---GAVQRYFLALEE : 578
DiinvUNTiw : YAG-YTIRRLCQEMHDFYKERKVNVLQKHLFLKNYFPIYSMSPQEANYELVRGHGELIDIAEAEGCVALEGGVPYPPGVPCIQPGEKWS---KTAIAYFMALAE : 578
VedisUNT-w : YKG-YHIRQLCQEMHDFYKSRNISLLQQRLFLGEYFPDYVMNPQEANFEFQRNKGELVPISEAEGRIALEGALPYPPGVLCVQPGERWS---KTACDYFLALEE : 578
LabacODCiw : YQG-YTIRQLCQEMHEYYAKNEIYKLQQDLFLKKTFQDYEMTPAEADKLFMKNEGELVDIDEIEGRVALEGALPYPPGVFIVAPGEKWQ---KIDVDYFKILMG : 573
GrbetUNT-w : YKN-VRLRDLCAEMHAFYRDRQVSRLQAEQFMAQHLPEMAMLPHEAMRELVRNNVDYLPIPEEAQGRIAATLWLVYPPGIATIVPGERLGPNAEPMVAYIKAFED : 609
TethUNT-a  : ---------------------------QELIYVQLTKKRKILTPLTKVMSS---DKEERKIQDCIGRISGNLQYSCPPGYPVITFGEEIR---KEHVEILGQNFL : 456
                                                           p  a                g         yPPG p    pGe

                *      640
StlugYaaOa : -DSGVR-VEGINNKKILVK- : 442
StaurYaaOa : -ETGMI-VEGIKNNKILVE- : 440
LimonYaaOa : -TRHYQGGEKLAENYIRVF- : 451
BasubYaaOa : MKTHVQGNMKIKEKQLLVYI : 472
CldifYaaOa : KGMIVSGLKDVDLNFIQVI- : 464
CrserUNT-a : HGADLQGIIDK-TARYVQVV : 476
CoproUNT-a : EGAEVQGTADP-ELEFIQVL : 477
CaexiODC-a : KGGLVQGSIRYGSEIAIRVV : 473
UTG1RsUNTa : KDIRVSGQETG-MLRTVKVF : 476
ElubaUNT-a : KKIRVSGQETE-SLKTIKVV : 479
MeoxyUNT-a : YGARIDGVLEL-DEPQIRVL : 476
LeferLDC-a : TGATLDG-LDS-DNRLINIV : 478
BacihADC-a : VGLPVQGPEDP-NLQYLRVI : 478
PesimADC-a : AGLPVQGPEDP-EIKHLRII : 477
BasubADC-a : AGLPVQGPEDS-TLHMIRVI : 477
FunucODC-a : ADLHIQGMEDP-ELETI--- : 475
FuulcUNT-a : AKLHIQGMADP-ELININVI : 478
IlpolUNT-a : AELHIQGMDDH-ELQYINVI : 477
FunecODC-a : AKLQLQGMEDS-ELTTINII : 478
ClbotODC-a : TGLYVQGTEDP-EVEYIKIV : 476
CltetUNT-a : TGLYVQGTEDL-EVNFIKVV : 476
ThethUNT-a : ANLSIQGTEDP-EVNYIKVV : 476
ThtheOLDCa : AKLSIQGTEDP-NVEYIKVV : 476
LebucUNT-a : KGCLLTGTEDM-HVEKINVV : 472
BrmurUNT-a : KGCVMQGTEDI-ELNNIQVM : 472
StpneADC-a : RGCSLQGTEDP-EVNHINVI : 472
SerumADC-a : KGCSLQGTQDP-ECREMRIL : 473
HutheADC-a : QKTQLQGTADP-YIDHIMVL : 473
PsychADC-a : QKAVITGMNDK-TLNNISIL : 470
CldifADC-a : SNAYLTDVQDK-NLDRILVI : 474
AclaiUNT-a : QGSTIL-SDT--EDGYIKVV : 470
MeblaADC-a : NSDCVQ-KDSK-EKDHILVV : 472
ChvarUNT-a : HGGTVTGCSDG-SLHTLRVV : 488
```

```
PhpatUNT-a : AGGAVSGAIDD-DFKSVRVL : 496
NopunUNT-a : MGGFISGCNDS-SLKTLKVV : 497
SyspeUNT-a : FWRDQIPDRLS-VVSGG--- : 468
PachrUNT-a : LWPMQIPSMIS-VIVE---- : 461
StcoeUNT-a : AGMYLPDPTDP-ALETVRVV : 479
MysmeUNT-a : AGMNLPDPADP-QMGSIRVM : 478
BoperODCcw : FNERFPGFETYIHGLA---- : 584
RalstODCcw : FNKLFPGFETDIHGLV---- : 592
LebifUNT-w : FNTKFPGFETDIHGLV---- : 582
PseaeLDCcw : FERAFPGFDSDVHGLQ---- : 586
EscoADCi-w : WDHHFPGFEHETEGT----- : 602
FuvaUNTi-w : WDKAFPGFEHETEGT----- : 588
MebooUNTiw : FDRRFPGFEHDTHGI----- : 605
StambUNT-w : FDRRFPGFGSETHGVTRAP- : 613
AcspeUNT-w : FGKRFPGFERETHGIE---- : 607
VivadUNTiw : FDKNFPGFEHEIQGVN---- : 607
EscoLDCi-w : IGAHYPGFETDIHGAY---- : 568
EscoLDCc-w : VGQHYPGFETDIHGAK---- : 568
PypisUNT-w : TGELFPGFDTEIHGAY---- : 568
FratuLDCcw : IGSMLPGFDTDIHGPE---- : 568
ElminUNT-w : FDNMFPGFSTEFHGVK---- : 591
StlugODCiw : GINKFPGFAPEIQGVY---- : 594
StepiODCiw : GINEFPGFAPEIQGVY---- : 594
OeoenODCiw : SINRFPGFAPEIQGVY---- : 594
EscoODCi-w : GINLLPGFAPELQGVY---- : 594
EscoODCc-w : GVNLLPGFSPELQGVY---- : 594
DiinvUNTiw : VFNLFPGFTPELQGVY---- : 594
VedisUNT-w : GINLLPGFAPEIQGVY---- : 594
LabacODCiw : AIDKFPGFDPEIQGVY---- : 589
GrbetUNT-w : AANQFPGFENEIQGLY---- : 625
TethUNT-a  : VEVLIEDY------------ : 464
                   g
```
